# IL-12 sensing in neurons induces neuroprotective CNS tissue adaptation and attenuates neuroinflammation in mice

**DOI:** 10.1038/s41593-023-01435-z

**Published:** 2023-09-25

**Authors:** Myrto Andreadou, Florian Ingelfinger, Donatella De Feo, Teresa L. M. Cramer, Selma Tuzlak, Ekaterina Friebel, Bettina Schreiner, Pascale Eede, Shirin Schneeberger, Maria Geesdorf, Frederike Ridder, Christina A. Welsh, Laura Power, Daniel Kirschenbaum, Shiva K. Tyagarajan, Melanie Greter, Frank L. Heppner, Sarah Mundt, Burkhard Becher

**Affiliations:** 1 Institute of Experimental Immunology, University of Zurich, Zurich, Switzerland; 2 Department of Neurology, University Hospital Zurich, Zurich, Switzerland; 3 Institute of Pharmacology and Toxicology, Neurodevelopmental Pharmacology, University of Zurich, Zurich, Switzerland; 4 Department of Neuropathology, Charité–Universitätsmedizin Berlin, corporate member of Freie Universität Berlin and Humboldt–Universität zu Berlin, Berlin, Germany; 5 Cluster of Excellence, NeuroCure, Berlin, Germany; 6 Present Address: Institute of Neuropathology, University Hospital Zurich, Zurich, Switzerland; 7 German Center for Neurodegenerative Diseases (DZNE) Berlin, Berlin, Germany; 8 Present Address: Department of Systems Immunology, Weizmann Institute, Rehovot, Israel; 9 Present Address: Department of Neuropathology, Charité–Universitätsmedizin Berlin, corporate member of Freie Universität Berlin and Humboldt–Universität zu Berlin, Berlin, Germany

**Keywords:** Cytokines, Multiple sclerosis

## Abstract

Interleukin-12 (IL-12) is a potent driver of type 1 immunity. Paradoxically, in autoimmune conditions, including of the CNS, IL-12 reduces inflammation. The underlying mechanism behind these opposing properties and the involved cellular players remain elusive. Here we map IL-12 receptor (IL-12R) expression to NK and T cells as well as neurons and oligodendrocytes. Conditionally ablating the IL-12R across these cell types in adult mice and assessing their susceptibility to experimental autoimmune encephalomyelitis revealed that the neuroprotective role of IL-12 is mediated by neuroectoderm-derived cells, specifically neurons, and not immune cells. In human brain tissue from donors with multiple sclerosis, we observe an IL-12R distribution comparable to mice, suggesting similar mechanisms in mice and humans. Combining flow cytometry, bulk and single-nucleus RNA sequencing, we reveal an IL-12-induced neuroprotective tissue adaption preventing early neurodegeneration and sustaining trophic factor release during neuroinflammation, thereby maintaining CNS integrity in mice.

## Abstract (web summary)

Interleukin-12 (IL-12) can have anti-inflammatory properties; however, the underlying mechanisms are unclear. Here, the authors show that IL-12-sensing neurons mediate IL-12-induced neuroprotective tissue adaptation in autoimmune conditions of the CNS.

## Main

Multiple sclerosis (MS) is a chronic inflammatory disease of the CNS characterized by cytokine dysregulation, demyelination and neuronal loss^1^. The inflammatory lesions in MS result from type 1 immunity, which drives leukocyte infiltration and phagocyte-mediated immunopathology^2^. The archetypical inducer of type 1 immunity is the cytokine IL-12, produced by antigen-presenting cells^3^. However, with the discovery of the closely related cytokine of the same superfamily, namely IL-23 (ref. ^4^), we and others found that IL-23 plays a nonredundant role in the development of CNS inflammation in preclinical models of MS^5,6^. Surprisingly, these studies also discovered that IL-12 ameliorated the disease and, therefore, has a regulatory immunosuppressive role in neuroinflammation. IL-12 and IL-23 are heterodimers harboring the unique subunits p35 and p19 engaging with IL-12Rβ2 and IL-23R, respectively, while sharing the common p40 subunit, which binds IL-12Rβ1. Dual targeting of IL-12 and IL-23 signaling, however, has not shown efficacy in a phase II clinical trial in MS therapy^7^, possibly due to interfering with two opposing underlying disease mechanisms simultaneously. In fact, higher *IL12RB2* expression is correlated with a lower risk of relapse in relapsing–remitting MS patients and better response to MS therapy^8^. Thus, IL-12 may attenuate neuroinflammation in MS not only in mice but also in humans. The mechanism behind IL-12-mediated tissue protection and immune regulation in MS has never been resolved.

To reveal the mechanistic underpinnings of how IL-12 limits immunopathology during CNS autoimmunity, we first identified and then conditionally targeted IL-12R-expressing cell types in neuroinflammation. We found that ablation of the IL-12R in the neuroectoderm (that is, astrocytes, neurons and oligodendrocytes) alone phenocopied the hypersusceptibility to experimental autoimmune encephalomyelitis (EAE) observed in germline *Il12rb2* knockout mice. We here describe the discovery of a neuroprotective loop triggered by IL-12-producing CNS-infiltrating monocyte-derived cells (MdCs), which implicated autocrine and paracrine survival and trophic factor release by distinct IL-12-sensing neuronal populations in neuroinflammation.

## Results

### Immune cells are dispensable for IL-12-mediated tissue protection

**Fig. 1:**
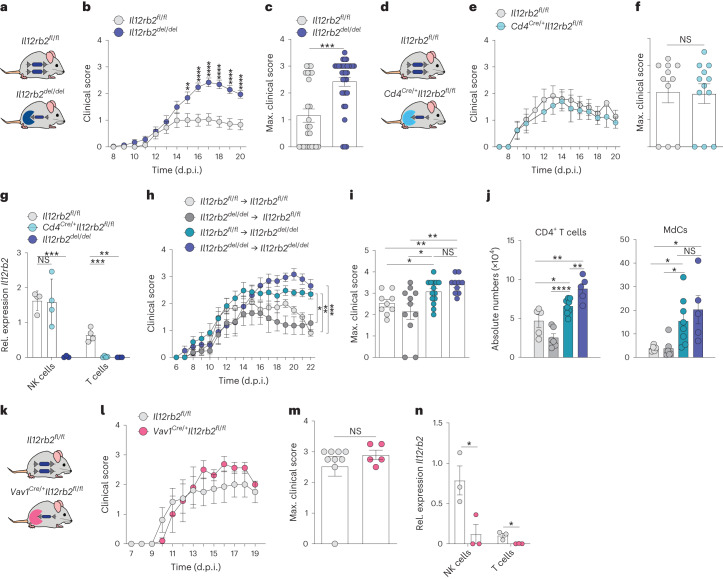
Leukocytes are dispensable for IL-12-mediated tissue protection in experimental autoimmune encephalomyelitis. **a**, Schematic illustrating *Il12rb2*^del/del^ and *Il12rb2*^fl/fl^ control mice. **b**,**c**, EAE course (**b**) and maximum EAE scores (**c**) in *Il12rb2*^del/del^ (*n* = 33, m/f) and *Il12rb2*^fl/fl^ mice (*n* = 25, m/f). Data were pooled from four experiments. Mixed-effects model, Sidak’s post hoc test (**P = 0.0063, ****P ≤ 0.0001, left to right) in **b**; two-tailed Mann–Whitney test (***P = 0.0001) in **c**. **d**, Schematic illustrating *Cd4*^Cre/+^*Il12rb2*^fl/fl^ and *Il12rb2*^fl/fl^ control mice. **e**,**f**, EAE course (**e**) and maximal EAE scores (**f**) in *Cd4*^Cre/+^*Il12rb2*^fl/fl^ (*n* = 12, m/f) and littermate controls (*n* = 11, m/f). Data were pooled from two experiments. Mixed-effects model, Bonferroni post hoc test (*P* > 0.9999) in **e**; two-tailed Mann–Whitney test (*P* = 0.6514*)* in **f**. **g**, *Il12rb2* mRNA relative expression in FACS-isolated CNS NK cells and CD3^+^ T cells of *Cd4*^Cre/+^*Il12rb2*^fl/fl^ (*n* = 4; f), littermate control *Il12rb2*^fl/fl^ (*n* = 4; f) and *Il12rb2*^del/del^ (*n* = 3; f) mice at 18 d.p.i. Data represent one experiment (unpaired two-tailed *t*-test, *P* = 0.9131; ***P = 0.002; ***P = 0.0006; **P = 0.0023, left to right). **h**,**i**, EAE course (**h**) and maximum EAE score (**i**) in bone marrow chimeras 6 weeks after immune reconstitution. Data were pooled from two experiments; *Il12rb2*^fl/fl ^→ *Il12rb2*^fl/fl^ (*n* = 9, m/f), *Il12rb2*^del/del ^→ *Il12rb2*^fl/fl^ (*n* = 11, m/f), *Il12rb2*^fl/fl ^→ *Il12rb2*^del/del^ (*n* = 14, m/f) and *Il12rb2*^del/del ^→ *Il12rb2*^del/del^ (*n* = 10, m/f). Two-way ANOVA, Bonferroni post hoc test (*P = 0.0234; **P = 0.0017, ***P ≤ 0.0005) in **h**; two-tailed Mann–Whitney test (*P = 0.0293; **P = 0.0011, *P = 0.0277; **P = 0.0023; *P* = 0.1855, left to right) in **i**. **j**, Absolute numbers of CNS CD45^+^CD44^+^CX3CR1^−^Ly6G^−^TCRβ^+^CD8^−^CD4^+^ T cells and CD45^+^CD44^+^CX3CR1^−^LY6G^−^TCRβ^−^NK1.1^−^SiglecF^−^Ly6C^+^CD11b^+^MHCII^+^ MdCs at 23 d.p.i. as measured by FC. Data are representative for two experiments; *Il12rb2*^fl/fl ^→ *Il12rb2*^fl/fl^ (*n* = 5, m/f), *Il12rb2*^del/del ^→ *Il12rb2*^fl/fl^ (*n* = 6, m/f), *Il12rb2*^fl/fl ^→ *Il12rb2*^del/del^ (*n* = 8, m/f) and *Il12rb2*^del/del ^→ *Il12rb2*^del/del^ (*n* = 5, m/f). Left graph: unpaired two-tailed *t*-test, *P = 0.0299; **P = 0.0049, ****P ≤ 0.0001; **P = 0.009, left to right; right graph: unpaired two-tailed *t*-test, *P = 0.0293; *P = 0.0261; *P = 0.0221; *P* = 0.4688; left to right. **k**, Schematic illustrating *Vav1*^Cre/+^*Il12rb2*^fl/fl^ and *Il12rb2*^fl/fl^ control mice. **l**,**m**, EAE course (**l**) and maximal EAE scores (**m**) in *Vav1*^Cre/+^*Il12rb2*^fl/fl^ (*n* = 5, m/f) and littermate controls (*n* = 9, m/f). Data were pooled from two experiments. Mixed-effects model, Bonferroni post hoc test (*P* > 0.9999) in **l**; two-tailed Mann–Whitney test (*P* = 0.6349) in **m**. **n**, *Il12rb2* mRNA expression of FACS-isolated CNS NK cells and CD3^+^ T cells of *Vav1*^Cre/+^*Il12rb2*^fl/fl^ (*n* = 3; m) mice and littermate controls (*n* = 3; m) at 19 d.p.i. (unpaired two-tailed *t*-test, *P = 0.0370; *P = 0.0224, left to right). Each symbol represents one animal. Data are shown as mean ± s.e.m. NS, not significant; m, male; f, female. Source data

**Extended Data Fig. 1:**
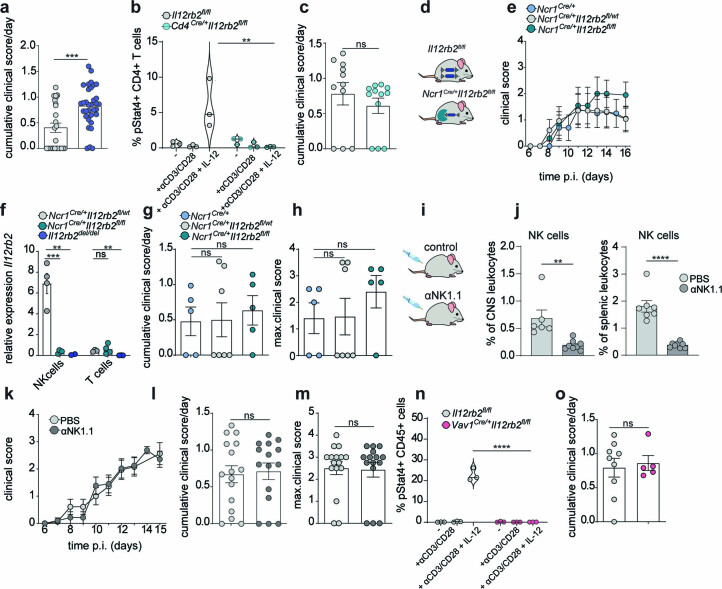
Loss of IL-12 signalling in leukocytes does not affect EAE outcome. (a) Cumulative EAE score/day in *Il12rb2*^*del/del*^ (n = 33, m/f) and *Il12rb2*^*fl/fl*^ mice (n = 25, m/f). Data are pooled from 4 experiments (unpaired two –tailed *t*- test, ***P = 0.0004). (b) % pSTAT4^+^ CD4^+^ splenocytes from steady-state *CD4*^*Cre/+*^*Il12rb2*^*fl/fl*^ and littermate controls (n = 3; m/f) determined by FC. Data are from one experiment (two-way ANOVA, Bonferroni post hoc test, *P* > 0.9999; *P* > 0.9999; **P = 0.0011, left to right). (c) Cumulative EAE score/day in *CD4*^*Cre/+*^*Il12rb2*^*fl/fl*^ (n = 12, m/f) and littermate controls (n = 11, m/f). Data are pooled from 2 experiments (unpaired two-tailed *t*-test, *P* = 0.3786). (d) Schematic for *Ncr1*^*Cre/+*^*Il12rb2*^*fl/fl*^ strain (e) EAE course in *Ncr1*^*Cre/+*^ (n = 5, f), *Nkp46*^*Cre/+*^*Il12rb2*^*fl/wt*^ (n = 7, f) and *Ncr1*^*Cre/+*^*Il12rb2*^*fl/fl*^ mice (n = 5, f). Data are pooled from two experiments (Mixed-effects model, Bonferroni post hoc test, *P* > 0.9999). (f) *Il12rb2* mRNA expression of FACS isolated NK cells and CD3^+^ splenocytes of *Ncr1*^*Cre/+*^ *Il12rb2*^*fl/fl*^ (n = 4), littermate *Ncr1*^*Cre/+*^ *Il12rb2*^*fl/wt*^ controls (n = 4) and *Il12rb2*^*del/del*^ (n = 3) mice at 17 dpi. Data represent one experiment (unpaired two-tailed *t*-test, ***P = 0.0005; **P = 0.0092; *P* = 0.6154; **P = 0.0011, left to right). (g) Cumulative EAE score/day (Unpaired two –tailed *t*- test (*P* = 0.9492; *P* = 0.606; *P* = 0.698, left to right)) and (h) maximal EAE score (two-tailed Mann-Whitney test (*P* = 0.8864*; P* = 0.0952*; P* = 0.8232, left to right) corresponding to (e). (i) C57BL/6 mice were treated with anti-NK1.1 depleting antibody or PBS at 0, 2, 8 and 14 dpi. (j) CNS and splenic NK cell depletion was assessed by FC at day 15 p.i. Shown is % live CD4^−^CD8^−^CD3^−^B220^−^Ly6G^−^CD44^+^CXCR1^−^CD49b^+^ NK cells in (n = 8, m/f) anti-NK1.1 and (n = 7, m/f) PBS-treated mice. Data represent one experiment (unpaired two –tailed *t*-test, **P = 0.0039; ****P < 0.0001, left to right). (k) Clinical EAE course, (l) cumulative EAE score/day and (m) maximal EAE score in anti-NK1.1- (n = 15, m/f) and PBS-treated mice (n = 16, m/f). Data are pooled from 2 experiments (Mixed-effects model, Bonferroni post hoc test (*P* > 0.9999) in (k); unpaired two-tailed *t*-test (*P* = 0.8076) in (l); two-tailed Mann-Whitney test (*P* = 0.8362) in (m). (n) %pSTAT4^+^ CD45^+^cells upon IL-12 stimulation of splenocytes from steady-state *Vav1*^*Cre/+*^*Il12rb2*^*fl/fl*^ and littermate controls determined by FC. Data represent one experiment (n = 3 per group) (two-way ANOVA, Bonferroni post hoc test, *P* > 0.9999; *P* > 0.9999; ****P < 0.0001, left to right). (o) Cumulative EAE score/day of *Vav1*^*Cre/+*^*Il12rb2*^*fl/fl*^ (n = 5, m/f) and littermate controls (n = 9, m/f). The data are pooled from 2 experiments (unpaired two–tailed *t*-test, *P* = 0.7473). Each symbol represents one animal. Data are shown as mean ± s.e.m. ns=not significant; m=male, f=female.

To identify the mechanism by which IL-12 limits immunopathology in CNS neuroinflammation, we first generated a conditional strain of IL-12-specific receptor subunit b2: *Il12rb2*^fl^ (ref. ^9^). We crossed *Il12rb2*^fl/fl^ mice with *CMV*^Cre^ mice to generate mice lacking *Il12rb2* in all tissues, hereafter termed *Il12rb2*^del/del^ mice (Fig. 1a). These mice were hypersusceptible to myelin oligodendrocyte glycoprotein (MOG)-induced EAE, as previously reported in mice lacking functional IL-12 (refs. ^5,6^; Fig. 1b,c). While the time to clinical disease onset was comparable between mice with or without *Il12rb2* (Fig. 1b), the absence of IL-12 signaling was associated with significantly higher maximal and cumulative EAE scores (Fig. 1c and Extended Data Fig. 1a).

As helper T (T_H_) cells are the main drivers of MOG-induced EAE, we asked whether deleting the IL-12R across aβ T cells would recapitulate the exacerbated clinical symptoms seen in *Il12rb2*^del/del^ mice. We crossed *Il12rb2*^fl/fl^ mice with *Cd4*^Cre/+^ mice (Fig. 1d–g), generating progeny in which *Il12rb2* was deleted in T cells. By analyzing IL-12-induced phosphorylation of STAT4 (Extended Data Fig. 1b) and qPCR (Fig. 1g), we confirmed the deletion of *Il12rb2* in T cells. Surprisingly, *Cd4*^Cre/+^*Il12rb2*^fl/fl^ mice and *Il12rb2*^fl/fl^ littermates developed EAE of similar clinical severity (Fig. 1f and Extended Data Fig. 1c) suggesting that T cells are not responsible for the IL-12-mediated tissue protection. Similarly, *Ncr1*^Cre/+^*Il12rb2*^*fl/fl*^ mice showed comparable clinical outcomes to the littermate control group suggesting no major contribution of natural killer (NK) cells to the IL-12-induced immunoregulatory circuit (Extended Data Fig. 1d–h).

As testing for equivalence was not conclusive for this dataset, we confirmed that NK cells had no measurable impact on EAE disease development, adding to the controversy of the role of NK cells in CNS autoimmunity^10^. Here, mice treated with an anti-NK1.1 antibody to delete NK cells showed similar clinical symptoms compared to the PBS-treated control group (Extended Data Fig. 1i–m). Together, our findings eliminate the two most obvious targets within the IL-12R-expressing lymphocyte compartment.

We next generated bone marrow chimeras in which *Il12rb2* was expressed either in the adult hematopoietic stem cell-derived systemic immune compartment or in radioresistant cells of the CNS (Table 1) (Fig. 1h–j). We found that the loss of IL-12R in immune cells did not affect the clinical outcome of EAE. In contrast, mice lacking *Il12rb2* expression in radioresistant cells phenocopied the *Il12rb2*^del/del^ strain (Fig. 1h–i) accompanied by significantly more infiltration of CD4^+^ T cells and MdCs into their CNS (Fig. 1j). This was further validated in *Vav1*^Cre/+^*Il12rb2*^fl/fl^ mice, which lack *Il12rb2* in all hematopoietic cells (and endothelial cells) but did not show exacerbated disease compared to littermate control mice (Fig. 1k–n and Extended Data Fig. 1n,o). Together, our findings allow the elimination of immune cells (including CNS-resident microglia^11^) from the list of potential candidates for the IL-12-mediated protective phenotype in EAE and indicate that the protective features of IL-12 are mediated by neuroectodermal cells.

### Neurons and oligodendrocytes sense IL-12 in mouse and human

**Fig. 2:**
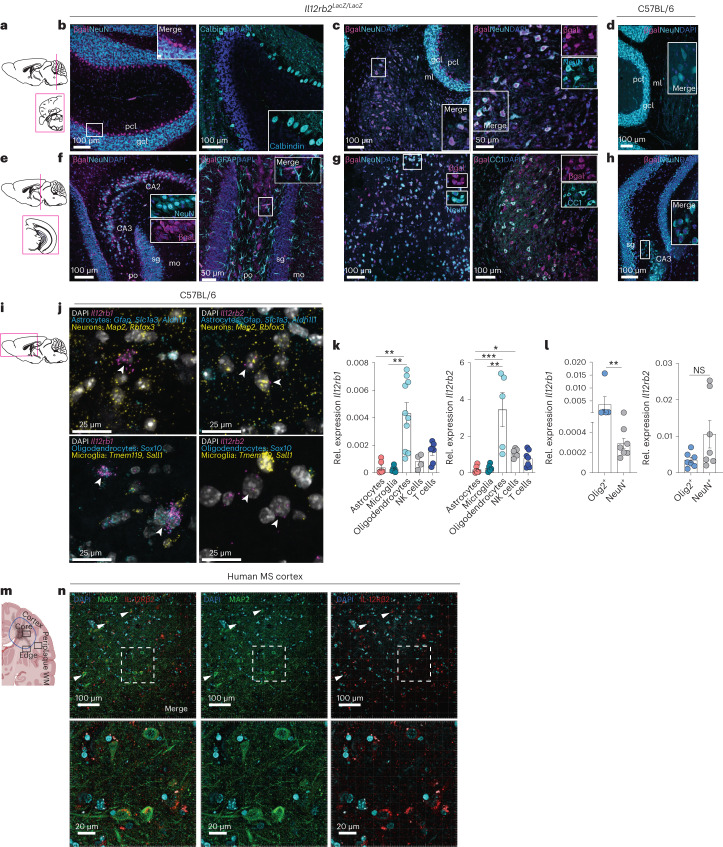
Neurons and oligodendrocytes are molecularly equipped to sense IL-12. **a**, Schematic of the mouse cerebellum showing the region represented by the following images. **b**–**d**, Immunostaining of β-gal^+^Calbindin^+^ and β-gal^+^NeuN^+^ cells in the steady-state cerebellum of *Il12rb2*^*LacZ/LacZ*^ reporter mice (*n* = 6 mice, m/f; data are representative for one of three experiments) and β-gal^−^NeuN^+^ cells in control C57BL/6 mice. **e**, Schematic of the mouse hippocampus showing region represented by the following images. **f**–**h**, Immunostaining of β-gal^+^NeuN^+^, βgal^−^GFAP^+^ and β-gal^+^ CC1^+^ cells in the hippocampal and cortical region of steady-state *Il12rb2*^*LacZ/LacZ*^ reporter mice (*n* = 5 mice, m/f; data are representative for one of three experiments) and β-gal^−^NeuN^+^ cells in control C57BL/6 mice. In **b**–**d** and **f**–**h**, scale bars indicate 50 μm or 100 μm. Insets are enlargements of outlined regions in the original images. **i**, Schematic of the mouse cerebrum showing the region represented by the following images. **j**, Multiplexed single-molecule RNA fluorescence in situ hybridization (RNAscope) in steady-state C57BL/6 mouse cerebral tissue showing *Il12rb2* gene coexpression in *Map2*^*+*^*Rbfox3*^*+*^ neurons and *Il12rb1* coexpression in *Sox10*^+^ oligodendrocytes. Scale bars, 25 μm. **k**, *Il12rb1* and *Il12rb2* mRNA expression of CD45^−^CD11b^−^O4^−^ACSA-2^+^astrocytes, CD45^lo^CD44^lo^CD11b^+^CX3CR1^+^ microglia, CD45^−^CD11b^−^ACSA-2^−^ O4^+^ oligodendrocytes, NK cells and CD3^+^ T cells isolated by FACS from the CNS of C57BL/6 mice at peak EAE (*n* = 4–10, m/f; number of dots represents the number of biologically independent replicates per sample; 14 d.p.i.). Data are shown as the mean ± s.e.m. Kruskal–Wallis test, corrected Dunn’s test (**P = 0.0012; ** *P* = 0.0016; ***P = 0.0009; *P = 0.0391; **P = 0.0052, left to right). **l**, *Il12rb1* and *Il12rb2* mRNA expression of Hoechst^+^ Olig2^+^ oligodendrocyte nuclei and Hoechst^+^ NeuN^+^ neuronal nuclei isolated by FANS from the CNS of C57BL/6 mice (*n* = 5–7; number of dots represents the number of biologically independent replicates per sample) at peak EAE (14 d.p.i.). Data are shown as mean ± s.e.m. (unpaired two-tailed Mann–Whitney test, **P = 0.0293, *P* = 0.1282, left to right). **m**, Schematic of human MS brain lesions (created with BioRender.com). **n**, Representative immunofluorescence of IL-12Rβ2-expressing MAP2^+^ neurons in brain tissue samples from individuals with MS (*n* = 3; data representative for one of three experiments). Scale bars, 20 μm and 100 μm. Lower images depict enlargements of outlined regions in the original (top) images. Gcl, granule cell layer; ml, molecular layer; pcl, Purkinje cell layer; sg (DG-sg), dentate gyrus, granule cell layer; po (DG-po), dentate gyrus, polymorph layer; mo (DG-mo), dentate gyrus, molecular layer; m, male; f, female. Source data

**Extended Data Fig. 2:**
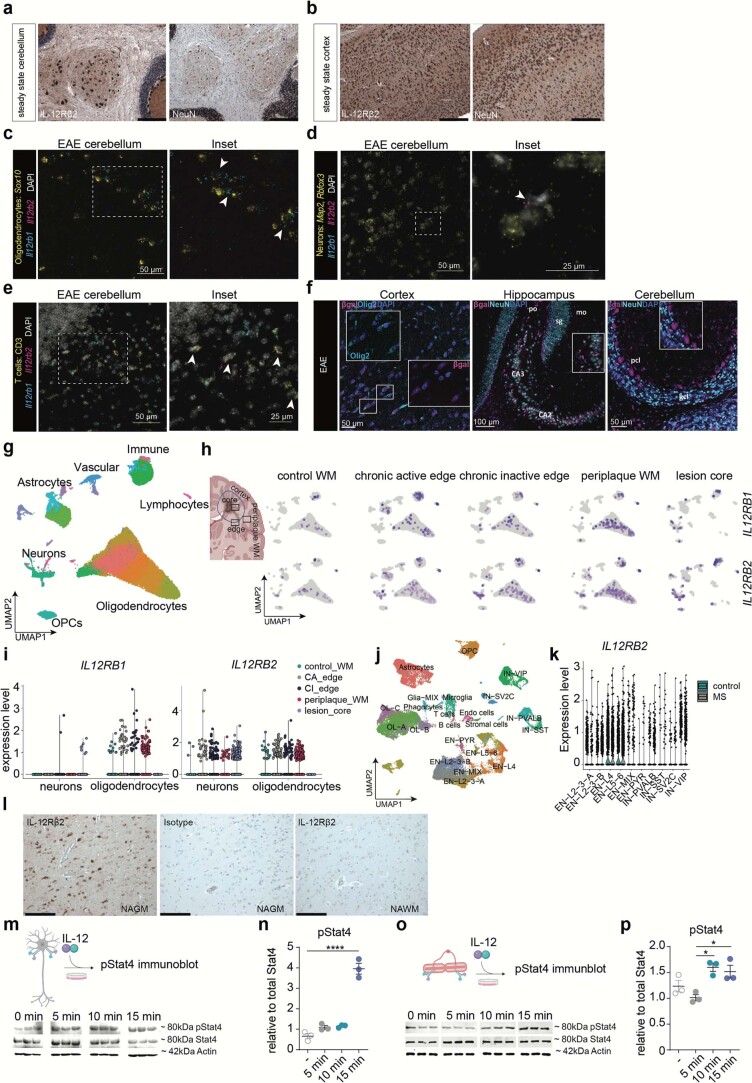
Murine and human neuroectodermal cells express *Il12rb2*. (a-b) Representative IHC for IL-12Rβ2^+^and NeuN^+^ cells in the steady-state C57BL/6 mouse brain (m/f; data are representative for 1 out of 3 experiments); scale bar= 200 μm. (c-e) Representative RNAscope® images of EAE C57BL/6 mouse brain. Cell markers were co-stained with probes for *Il12rb1* and *Il12rb2*, as highlighted by arrowheads. Scale bars = 25 μm (insets) or 50 μm. (f) Immunostaining of β-gal^+^Olig2^+^ and β-gal^+^NeuN^+^ cells in EAE *Il12rb2*^*LacZ/LacZ*^ reporter mice (n = 4, m/f; data are representative for 1 out of 3 experiments). Scale bars = 50 μm or 100 μm. Insets are enlargements of outlined regions in the original images. GCL, granule cell layer; PCL, Purkinje cell layer; sg (DG-sg), dentate gyrus, granule cell layer; po (DG-po), dentate gyrus, polymorph layer; mo (DG-mo), dentate gyrus, molecular layer. (g) snRNA-seq of n = 66,432 nuclei from white matter (WM) areas of five patients with progressive MS and three age- and sex-matched non affected, non-dementia controls (accessed at GSE180759 (ref. ^13^). UMAP displaying identified cell clusters, sorted by cell type (OPCs: oligodendrocyte progenitor cells). (h) Schematic illustration of MS lesion (left). *IL12RB1*- and *IL12RB2*-expression overlayed on UMAP, sorted by pathological condition (right). (i) Violin plots displaying *IL12RB1* and *IL12RB2* transcripts in neurons and oligodendrocytes, split by pathological condition (violin plots depict the number of counts per cell across type/site of lesions ; Wilcoxon rank sum test). (j) snRNAseq of n = 39,579 nuclei from whole MS tissue sections (n = 12) and control brain tissue (n = 9) (accessed at PRJNA544731 (ref. ^14^). UMAP with identified cell clusters, sorted by cell type (EN: excitatory neurons; IN: interneurons; PVALB: parvalbumin; L: layer). (k) Violin plots depicting neuronal *IL12RB2* transcripts, split by pathological condition (see above; Wilcoxon rank sum test). (l) Brain sections from MS patients were stained for IL-12Rβ2 or total rabbit IgG for IHC (n = 3; data are representative for 1 out of 3 experiments); scale bar= 200 μm [normal appearing grey matter (NAGM), normal appearing WM (NAWM)]. (m) Whole cell protein lysates were prepared from dissociated mouse cerebellar neurons at DIV 14 following 5-, 10- and 15-min stimulation with IL-12 (100 ng/mL), followed by western blot for total Stat4 and pStat4 (Tyr693) (loading control: actin) (n = 3 per group per time point). (n) Fold change in pSTAT4 (relative to total Stat4) protein expression. (o) Whole cell protein lysates were prepared from mouse primary oligodendrocyte cultures at DIV 4 following 5-, 10- and 15-min stimulation with IL-12 (100 ng/mL); western blot analysis was performed as in (m) (n = 3 per group per time point). (p) Quantification of pSTAT4 (relative to total Stat4). In (n) and (p) data are shown as mean ± SD and are representative for 1 out of 3 experiments. Statistical significance was evaluated by one-way ANOVA with Bonferroni multiple comparison post-hoc test (****P < 0.0001; *P = 0.0157; *P = 0.0371, left to right). Source data

We next sought to capture and localize potential sensors of IL-12, resident to the murine CNS. To do so, we used immunostaining for β-galactosidase (β-gal) to detect cells expressing IL-12Rβ2 protein within the steady-state CNS of *Il12rb2*^LacZ/LacZ^ reporter mice, coupled with multiplexed RNA fluorescence in situ hybridization (RNAscope) for *Il12rb1* and *Il12rb2* mRNA. IL-12R was expressed in the neuroectoderm, specifically in neurons and oligodendrocytes, already at steady state (Fig. 2a–h and Extended Data Fig. 2a,b), consistent with a recently published single-nucleus RNA-sequencing (snRNA-seq) dataset of adult mouse hippocampus^12^. Both β-gal/IL-12Rβ2 immunoreactivity (Fig. 2a–h and Extended Data Fig. 2a,b) and *Il12rb2* mRNA transcripts (Fig. 2i,j) were localized to NeuN-positive and *Rbfox3/Map2-*positive neuronal cells, respectively. The β-gal-positive signal was also present in CC1^+^ myelin-forming oligodendrocytes and mature oligodendrocytes (MOLs; Fig. 2g). While *Ιl12rb1* mRNA was more strongly expressed in *Sox10*-positive oligodendrocytes (Fig. 2i,j), neither astrocytes nor microglia expressed the IL-12R subunits, suggesting their inability to sense IL-12 (Fig. 2f,j). *Il12rb1/Il12rb2* gene expression patterns were similar in the inflamed murine CNS (Extended Data Fig. 2c–f) where we further validated our findings by qPCR of populations subjected to fluorescence-activated cell sorting (FACS; Fig. 2k) and fluorescence-activated nuclei sorting (FANS; Fig. 2l). Apart from infiltrating T cells and NK cells, IL-12R subtypes were strongly expressed on oligodendrocytes and neurons, thereby representing the primary radioresistant non-hematopoietic CNS-resident cells expressing the IL-12R complex at steady state and during MOG-induced neuroinflammation.

We next evaluated the presence of the IL-12-sensing molecular machinery in the human CNS by examining snRNA-seq datasets consisting of 66,432 nuclei from white matter (WM) lesional tissue of deceased individuals with progressive MS and age-matched and sex-matched controls who had died from non-neurological causes^13^ (Extended Data Fig. 2g–i). Analogous to the mouse, human neurons and oligodendrocytes expressed transcripts encoding IL-12R subtypes (Extended Data Fig. 2h,i). We next interrogated a second independent human dataset (*n* = 39,579 nuclei) of whole MS tissue sections^14^ (comprising cortical gray matter (GM), demyelinated lesions and underlying subcortical WM) of individuals with secondary progressive MS (*n* = 12) and controls (*n* = 9; Extended Data Fig. 2j,k). *IL12RB2* was expressed across all excitatory neuron clusters (upper, intermediate and deep cortical layers) at similar levels in MS and control samples (Extended Data Fig. 2k). We further confirmed neuronal IL-12Rβ2 protein expression in the cortex of individuals with MS by immunohistochemistry (Extended Data Fig. 2l). Finally, when we exposed primary mouse neurons (Extended Data Fig. 2m,n) or oligodendrocytes (Extended Data Fig. 2o,p) to recombinant IL-12, we observed a rapid increase of STAT4 phosphorylation—a hallmark of IL-12 receptor signaling. Together, these data demonstrate that in both mice and humans CNS-endogenous neuroectodermal cells are capable of sensing and transducing IL-12 signaling intracellularly.

### Neuroectodermal IL-12R signaling attenuates neuroinflammation

**Fig. 3:**
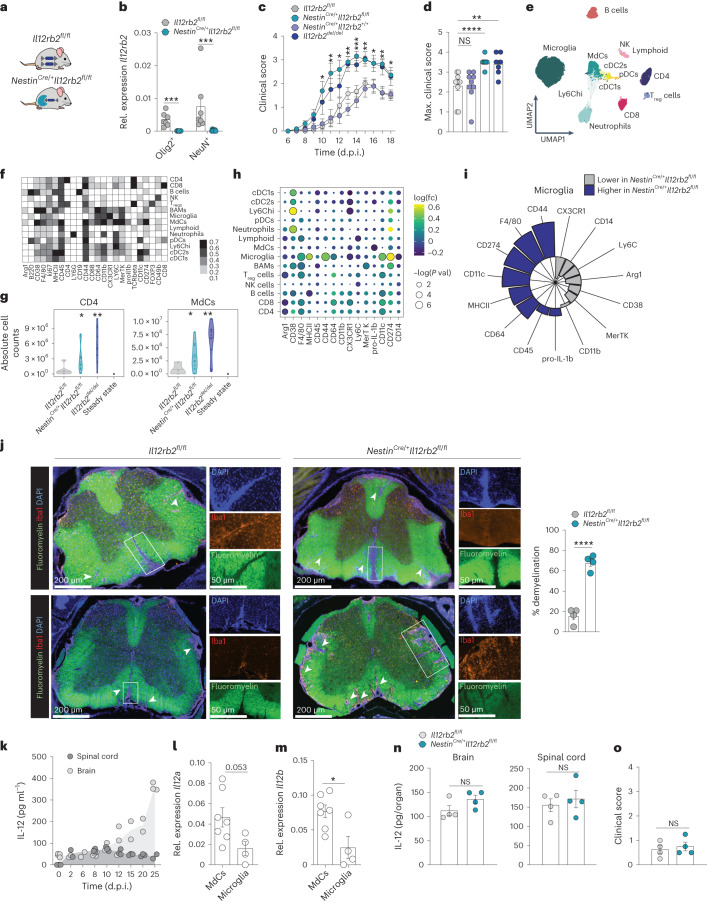
Neuroectodermal IL-12-sensing attenuates CNS immunopathology. **a**, Schematic of *Nestin*^Cre/+^*Il12rb2*^fl/fl^ and *Il12rb2*^fl/fl^ control mice. **b**, *Il12rb2* expression in FANS-isolated Hoechst^+^Olig2^+^ oligodendrocyte and Hoechst^+^NeuN^+^ neuronal nuclei from *Nestin*^Cre/+^*Il12rb2*^fl/fl^ (*n* = 7, m/f) and *Il12rb2*^fl/fl^ control mice (*n* = 7, m/f) at peak EAE (13 d.p.i.). Data are pooled from two experiments (two-tailed Mann–Whitney test, ***P = 0.0006; ***P = 0.0006, left to right). **c**,**d**, EAE course (**c**) and maximal EAE scores (**d**) of *Nestin*^Cre/+^*Il12rb2*^fl/fl^ (*n* = 8, m/f), *Il12rb2*^fl/fl^ littermate controls (*n* = 10, m/f), *Il12rb2*^del/del^ (*n* = 7, m/f) and *Nestin*^Cre/+^*Il12rb2*^+/+^ (*n* = 9, m/f) mice. Data were pooled from two experiments. Mixed-effects model with Bonferroni post hoc test between *Il12rb2*^fl/fl^ and *Nestin*^Cre/+^*Il12rb2*^fl/fl^ mice (*P = 0.0209; **P = 0.0045; *P = 0.0288; **P = 0.0046; ***P = 0.0005; **P = 0.0092; *P = 0.0221; **P = 0.0014; *P = 0.0131; left to right) in **c** and two-tailed Mann–Whitney test (*P* > 0.9999; ****P < 0.0001; **P = 0.0022, left to right) in **d**. **e**, UMAP of 60,000 cells normalized to sample size and proportional to the absolute cell number per group of *Nestin*^Cre/+^*Il12rb2*^fl/fl^ (*n* = 11, m/f), *Il12rb2*^fl/fl^ (*n* = 7, m/f), *Il12rb2*^del/del^ (*n* = 9, m/f) at peak EAE (13 d.p.i.). **f**, Heatmap depicting median marker expression per cluster. **g**, Cell counts of CNS CD4^+^ T cells and MdCs corresponding to **e**. Data were pooled from two experiments (unpaired two-tailed *t*-test; *P = 0.03681; **P = 0.00203; *P = 0.02085; **P = 0.00151, left to right). **h**, Dot plot depicting fold change expression and *P* value of the indicated markers per population. **i**, Cohen’s *d* effect size for the expression of the indicated activation molecules in microglia from *Nestin*^Cre/+^*Il12rb2*^*fl/fl*^ and *Il12rb2*^fl/fl^ mice. **j**, Representative WM staining with FluoroMyelin Green dye (top, thoracic; bottom, lumbar) and the percentage of myelin loss quantification in axial spinal cord sections at 19 d.p.i. in *Nestin*^Cre/+^*Il12rb2*^fl/fl^ and control mice (*n* = 4 mice per group). Insets highlight active, demyelinating inflammatory lesions with accumulation of DAPI^+^Iba1^+^ phagocytes. Individual lesions are pointed out by arrowheads. Scale bars, 50 μm and 200 μm. Unpaired two-tailed *t*-test, ****P < 0.0001). **k**, Kinetics of IL-12 (IL-12/23p40) concentration (ELISA) in the brain and spinal cord in C57BL/6 mice *n* = 2–5 per time point in EAE, m/f; number of dots represents the number of biologically independent replicates per sample. **l**,**m**, *Il12a* (**l**) and *Il12b* (**m**) mRNA expression in FACS-isolated CNS CD45^+^CD44^+^CD11b^+^Ly6G^−^Ly6C^+^ MdCs (*n* = 7) and CD45^lo^CD44^lo^CD11b^+^CX3CR1^+^ microglia (*n* = 4) of C57BL/6 mice (m/f) at disease onset (10 d.p.i.). Unpaired two-tailed *t*-test, *P* = 0.053; *P = 0.0137, left to right. **n**,**o**, IL-12 protein in brain and spinal cord lysates (**n**) and clinical score of *Nestin*^Cre/+^*Il12rb2*^fl/fl^ (*n* = 4, m) and littermate control mice (*n* = 4; m) at 10 d.p.i. (**o**). Two-tailed Mann–Whitney test, *P* = 0.1335; *P* = 0.5916; *P* = 0.7429; left to right). Each symbol represents one animal. Data are shown as the mean ± s.e.m.; m, male; f, female. Source data

**Extended Data Fig. 3:**
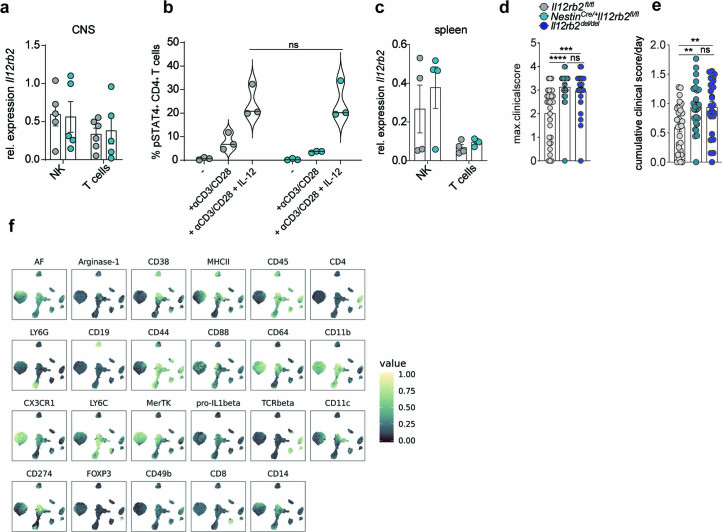
IL-12 engagement in neuroectodermal cells protects against neuroinflammation. (a) *Il12rb2* mRNA expression in NK cells and CD3^+^ T cells isolated by FACS from the CNS of MOG/CFA immunized *Nestin*^*Cre/+*^*Il12rb2*^*fl/fl*^ (n = 5, m/f) and littermate controls (n = 6, m/f) at 17 dpi. Pooled data from two independent experiments shown as mean ± s.e.m. (unpaired two –tailed *t*- test, *P* = 0.9009; *P* = 0.8027, left to right). (b) Frequency of pSTAT4^+^ CD4^+^cells upon IL-12 stimulation of splenocytes from steady-state *Nestin*^*Cre/+*^*Il12rb2*^*fl/fl*^ (n = 3, m) mice and littermate controls (n = 3, m). Data represent one experiment shown as mean ± s.e.m. (two-way ANOVA, Bonferroni’s post hoc test, *P* > 0.9999; *P* = 0.8631; *P* > 0.9999, left to right). (c) *Il12rb2* mRNA expression in NK cells and CD3^+^ T cells isolated by FACS from the spleen of *Nestin*^*Cre/+*^*Il12rb2*^*fl/fl*^ and littermate controls at late stage EAE (n = 4 per group, m/f). Data represent one experiment shown as mean ± s.e.m. unpaired two –tailed *t*- test, *P* = 0.5251; *P* = 0.1749, left to right). (d) Maximum disease scores and (e) cumulative clinical score/day of MOG-CFA immunized *Nestin*^*Cre/+*^*Il12rb2*^*fl/fl*^ (n = 26, m/f), littermate *Il12rb2*^*fl/fl*^ control (n = 30, m/f) and *Il12rb2*^*del/del*^ (n = 24, m/f) mice. Data are pooled from 6 independent experiments and represent mean ± s.e.m. Two-tailed Mann-Whitney test (****P < 0.0001; ***P = 0.0003; *P* = 0.4211, left to right) in (d); unpaired two –tailed *t*- test (**P = 0.0018; **P = 0.0054; *P* = 0.9615, left to right) in (e). (f) Marker expression overlaid onto UMAP of CNS leukocytes from *Nestin*^*Cre/+*^*Il12rb2*^*fl/fl*^ (n = 11, m/f), *Il12rb2*^*fl/fl*^ (n = 7, m/f), *Il12rb2*^*del/del*^ (n = 9, m/f) (related to Fig. 3e). ns= not significant. m, male; f, female.

We then asked whether the ability of the neuroectoderm to sense and respond to IL-12 was functionally linked to IL-12-induced tissue protection in EAE. We crossed *Il12rb2*^fl/fl^ mice to a *Nestin*^Cre/+^ strain (Fig. 3a). The *Nestin*^Cre/+^ strain permits the simultaneous targeting of both neurons and oligodendrocytes, which we identified as the relevant IL-12 receptor-bearing cell types in the CNS. Taking into account previous studies raising concerns about the targeting promiscuity of the *Nestin*^Cre/+^ strain outside the neuroectoderm^15^, we confirmed that ablation of *Il12rb2* occurred in neurons and oligodendrocytes (Fig. 3b) but not in splenic and CNS-infiltrating T cells and NK cells (Extended Data Fig. 3a–c). Strikingly, *Nestin*^Cre/+^*Il12rb2*^fl/fl^ mice phenocopied the EAE hypersusceptibility of the *Il12rb2* full knockout strain (Fig. 3c,d and Extended Data Fig. 3d,e). We excluded potential effects of *Cre* recombinase expression^15^ on the EAE outcome using *Nestin*^Cre/+^*Il12rb2*^+/+^ mice (Fig. 3c,d).

We next compared the immune cell landscape between *Nestin*^Cre/+^ *Il12rb2*^fl/fl^ and *Il12rb2*^fl/fl^ littermate control mice at peak disease using flow cytometry (FC) of CNS leukocytes (gated on live CD45^+^ singlets), coupled with dimensionality reduction (uniform manifold approximation and projection (UMAP)) and FlowSOM clustering (Fig. 3e–i and Extended Data Fig. 3f). We observed a significantly higher CNS infiltration of CD4^+^ T cells and MdCs (Fig. 3g), as well as a reactive microglia signature (Fig. 3h,i), consistent with the exacerbated EAE phenotype in the absence of IL-12R signaling in cells of the neuroectoderm. Quantitative immunofluorescence confirmed severe inflammation and CNS demyelination in *Nestin*^Cre/+^*Il12rb2*^fl/fl^ mice (Fig. 3j). Together, our data indicate that IL-12 signaling by cells of the neuroectoderm drives immunomodulatory and/or tissue-protective processes that attenuate inflammatory demyelinating CNS pathology in EAE.

### IL-12 induces neuroprotection and trophic factor release

We next sought to precisely disentangle the molecular underpinnings of this yet-unperceived neuroimmune cross-talk instructed by IL-12. Importantly, for this analysis, we opted for a time point when IL-12 is already present in the CNS but has not (yet) translated in clinical differences between *Nestin*^Cre/+^*Il12rb2*^fl/fl^ and *Il12rb2*^fl/fl^ control mice. Hence, we measured the concentration of IL-12 in brain and spinal cord lysates during EAE induction and disease (Fig. 3k). We found that IL-12 was barely detectable for almost a week after immunization, but began to increase from the day of clinical onset (10 days post immunization (d.p.i.); Fig. 3k), likely coinciding with the influx of MdCs. In fact, compared to microglia, infiltrating MdCs were the dominant source of IL-12 in the inflamed CNS (Fig. 3l,m). As fragile neuroectodermal cells are typically underrepresented in most single-cell omic approaches, we performed snRNA-seq of FANS-isolated Hoechst^+^ nuclei from the cerebellum, brainstem and cervical spinal cord of *Nestin*^Cre/+^*Il12rb2*^fl/fl^ mice and their *Il12rb2*^fl/fl^ littermates (*n* = 4 per group) at disease onset (10 d.p.i.), where clinical symptoms and also CNS protein levels of IL-12 were comparable between the two genotypes (Fig. 3n,o).

**Fig. 4:**
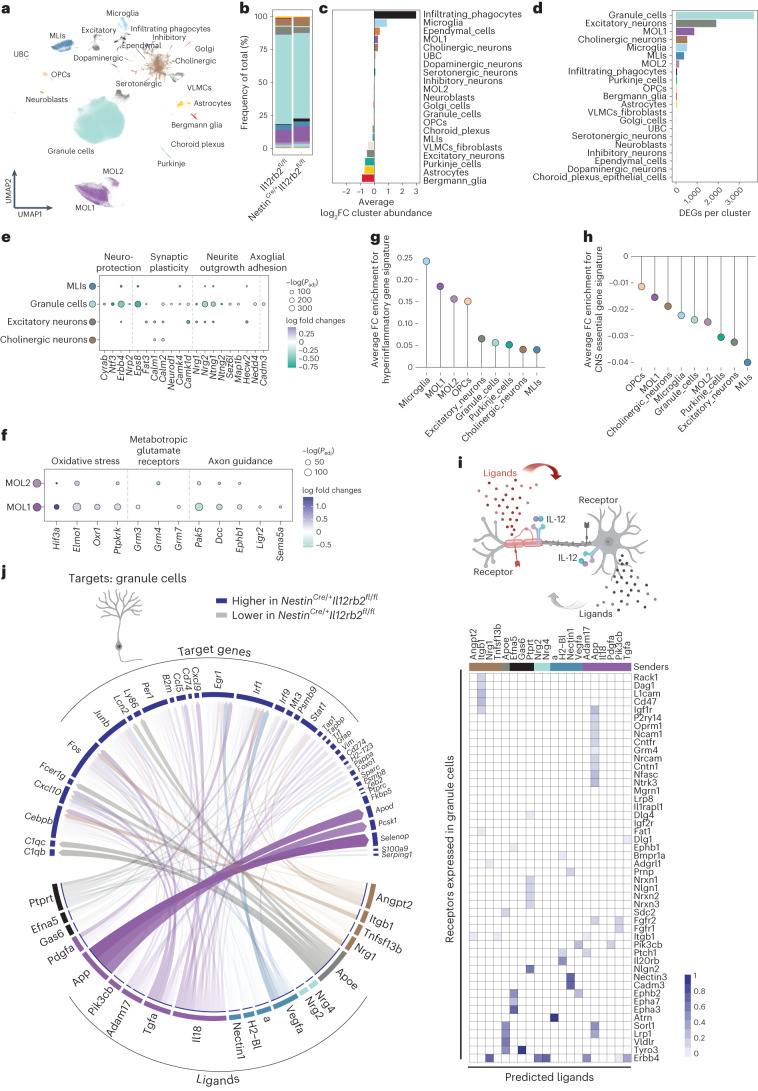
IL-12 signaling promotes neuronal survival, homeostasis and trophic support to oligodendrocytes in the inflamed murine CNS. Nuclei were isolated from pooled cerebellum, brainstem (dissected from one brain hemisphere) and cervical spinal cord from *Il12rb2*^fl/fl^ and *Nestin*^Cre/+^*Il12rb2*^fl/fl^ male mice (*n* = 4 per genotype) at the onset of EAE (10 d.p.i.). Nuclei were lysed, purified by FANS selecting for the Hoechst^+^ fraction and used for snRNA-seq. **a**, snRNA-seq clustering of 133,151 nuclei by cell type labeled on the basis of known lineage markers and visualized as a UMAP. Each dot corresponds to a single nucleus and each color to a cell-type cluster. **b**–**d**, Mean frequencies of cell clusters (**b**), average fold change of cluster abundance (**c**) and number of DEGs per cluster (**d**) between *Nestin*^Cre/+^*Il12rb2*^fl/fl^ and *Il12rb2*^fl/fl^ mice. **e**,**f**, Dot plots showing differential expression of hallmark genes across neurons and oligodendrocytes. Dot size indicates significance and color indicates log fold change between *Nestin*^Cre/+^*Il12rb2*^fl/fl^ and *Il12rb2*^fl/fl^ mice in the respective cell types. **g**,**h**, Average fold change of manually selected gene signatures for hyperinflammation and neuroprotection (Supplementary Table 2) between *Nestin*^Cre/+^*Il12rb2*^fl/fl^ and *Il12rb2*^fl/fl^ mice across cell types. **i**, Schematic of the receptor–ligand interaction analysis (NicheNet; created with BioRender.com). Senders were defined by their ability to sense IL-12 (expression of *Il12rb1* and/or *Il12rb2* transcripts) and the magnitude of their differential gene expression (cutoff > 35 DEGs). Both autocrine and paracrine ligand–receptor interactions were considered. **j**, Circos plots showing links between unique ligands from senders (ribbon color indicates subcluster of origin for each ligand; multiple sender cells are indicated by black ribbon) and predicted associated DEGs in granule cells. Transparency indicates interaction strength, and the ribbon thickness is proportional to the ligand’s regulatory potential. Heatmap (right) displaying potential receptors expressed in granule cells associated with each predicted ligand. Differential gene expression in **e**–**h** was tested using a two-sided Wilcoxon rank-sum test and applying a Benjamini–Hochberg correction and is presented in Supplementary Table 1. OPC, oligodendrocyte progenitor cell; VLMC, vascular and leptomeningeal cell. UBC, unipolar brush cells.

**Extended Data Fig. 4:**
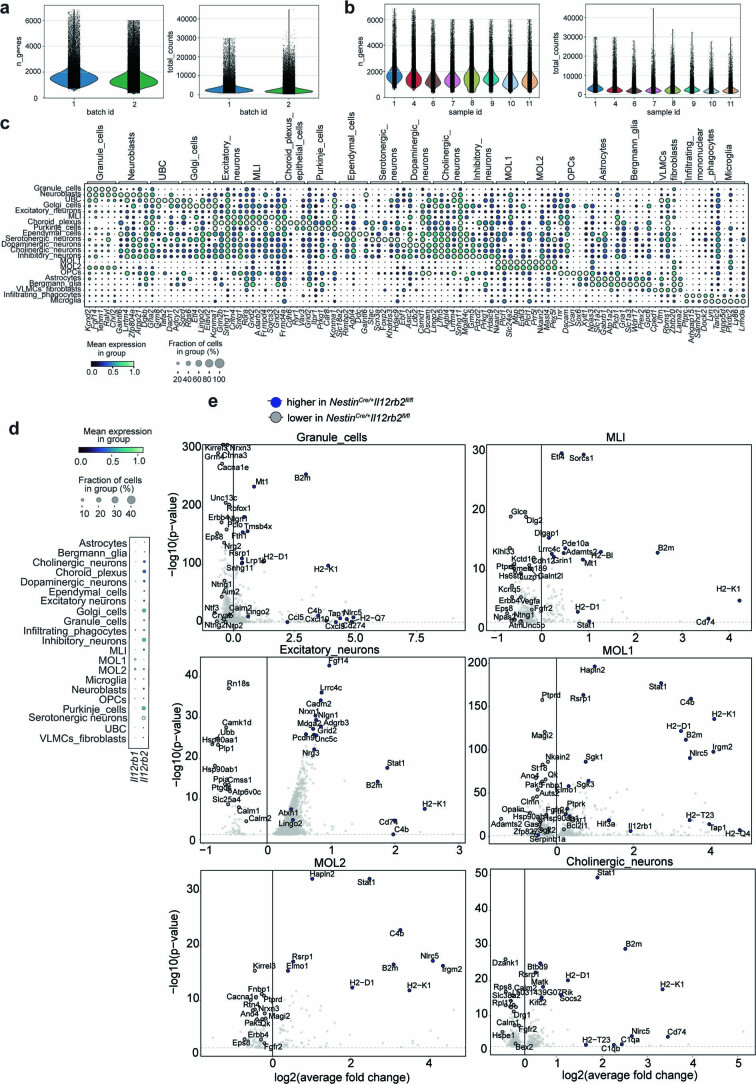
Direct IL-12 engagement of neuronal cells mediates neuroprotection. (a-b) Nuclei were isolated from the CNS of *Il12rb2*^*fl/fl*^ and *Nestin*^*Cre/+*^*Il12rb2*^*fl/fl*^ male mice (n = 4 per genotype) at the onset of EAE (10 dpi). The Hoechst^+^ fraction was FANS-selected and used for single-nucleus RNA sequencing. Violin plots depicting the distribution of (a) number of unique genes and total UMI counts per nuclei for each batch and (b) number of unique genes and total UMI counts detected in each sample linked to Fig. 4a. (c) Dot plot depicting the top 5 differentially expressed genes (DEGs) for each cluster and associated cluster labelling. Dot size corresponds to the percentage of nuclei expressing marker genes across all clusters and the colour shows the average expression level. (d) Dot plots depicting the expression of *Il12rb1* and *Il12rb2* for each cluster and associated cluster labelling. Dot size corresponds to the percentage of nuclei expressing the gene in each cluster, and the colour represents the average expression level. (e) Volcano plots displaying genes that are up- (blue) or downregulated (grey) in the inflamed *Nestin*^*Cre/+*^*Il12rb2*^*fl/fl*^ CNS for the highlighted neuronal or oligodendrocyte clusters. Dashed lines indicate significance thresholds (adjusted p-value < 0.05) used when identifying DEGs. Differential gene expression in (e) was tested using a two-sided Wilcoxon rank sum test and applying a Benjamini-Hochberg correction and is presented in Supplementary Table 1.

After quality control, doublet exclusion and batch integration, snRNA-seq yielded a total of 133,151 single-nucleus transcriptomic profiles, among which 1,453 median genes per nucleus and 2,428 median unique molecular identifiers (UMIs) per nucleus were detected (Fig. 4 and Extended Data Fig. 4a,b). Community detection of the combined dataset yielded 21 clusters that were assigned to diverse neuronal, glial and other cell types on the basis of known lineage marker genes (Fig. 4a,b and Extended Data Fig. 4c). Among these clusters were 5 populations of neurons reflecting the cellular composition of the cerebellum^16^ and 5 additional neuronal clusters classified by neurotransmitter type. We also captured 6 clusters of glial cells corresponding to oligodendrocytes, astrocytes, microglia, oligodendrocyte precursor cells and Bergmann glia. Non-neural cells encompassed infiltrating mononuclear phagocytes, vascular and leptomeningeal cells, ependymal cells, choroid plexus epithelial cells and neuroblasts. Among all recovered nuclei, 85% were neurons, and 15% were glial cells (Fig. 4b), consistent with previous descriptions^16,17^. When comparing cluster abundance between the two genotypes, we noted an increased frequency of both resident and infiltrating mononuclear phagocytes in *Nestin*^Cre/+^*Il12rb2*^fl/fl^ mice (Fig. 4c).

When we looked at the distribution of *Il12rb2* mRNA transcripts across the defined cell clusters, we found that it was most abundant in neuronal populations (Extended Data Fig. 4d), while *Il12rb1* transcripts were enriched within MOL subsets, confirming our initial observations. Next, we assessed how the transcriptional profile of the neuroectoderm differed in the presence or absence of IL-12 receptor signaling in EAE by interrogating differentially expressed genes (DEGs). This revealed pronounced transcriptional alterations particularly in granule cells, excitatory neurons, MOL1, cholinergic neurons, microglia, molecular layer interneurons (MLIs) and MOL2, suggesting a dominating action of IL-12 on these populations (Fig. 4d–f, Extended Data Fig. 4e and Supplementary Table 1).

The most abundant (and most affected) neuronal population—granule cells—showed reduced expression of genes involved in neuronal survival and neuroprotection (*Cryab*, *Ntf3*, *Erbb4*, *Nrp2*) in *Nestin*^Cre/+^*Il12rb2*^fl/fl^ mice (Fig. 4e, Extended Data Fig. 4e and Supplementary Table 1). Two examples are *Cryab*, an anti-apoptotic chaperone, which ameliorates EAE upon administration^18^ and AIM2 inflammasome signaling, which protects neurons from genotoxic stress and contributes to homeostatic CNS sculpting^19^ (Extended Data Fig. 4e). A similar pattern emerged in MLIs and cholinergic neurons (Fig. 4e and Extended Data Fig. 4e). In addition, we found that several genes implicated in synaptic plasticity (*Erbb4*, *Eps8*, *Fat3*, *Calm1*, *Calm2*, *Neurod1*, *Camk4* and *Camk1d*), neurite outgrowth/regeneration and guidance (*Nrg1*, *Nrg2*, *Ntng1*, *Ntng2*, *Sez6l*, *Map1b*, *Dab1*, *Hecw2* and *Nedd4l)*, as well as axoglial adhesion molecules required for proper targeting of myelin to axons (that is *Cadm3*)^20^ were expressed in neurons in an IL-12-dependent manner (Fig. 4e, Extended Data Fig. 4e and Supplementary Table 1). Together, these data implicate IL-12 signaling in promoting neuronal survival and homeostasis and antagonizing neuronal degeneration.

In addition to inducing neuroprotective features, IL-12 suppressed several genes linked to immune cell trafficking to the CNS, including *Ccl2*, *Ccl5*, *Ccl25*, *Cxcl9* and *Cxcl10* (Extended Data Fig. 4e and Supplementary Table 1) in granule cells. This could explain how neuroectodermal IL-12 signaling counteracts the recruitment of inflammatory infiltrates to the CNS.

Moreover, in MOL1 and MOL2 clusters, genes involved in cellular responses to oxidative cellular stress and programmed cell death (*Hif3a*, *Elmo1*, *Oxr1*, *Ptprk)* were suppressed by IL-12 (Fig. 4f, Extended Data Fig. 4e and Supplementary Table 1), while genes encoding metabotropic glutamate receptors (*Grm3*, *Grm4*, *Grm7*) and genes linked to axon guidance (*Pak5*, *Dcc*, *Ephb1*, *Lrig2*, *Sema5a*) were induced, indicating that IL-12 is required to maintain a physiological communication between MOLs and neuronal cells within the oligodendrocyte–axon unit.

Of note, an enrichment in genes involved in major histocompatibility complex (MHC) class I signaling (*B2m*, *H2-K1*, *H2-D1*, *H2-Q7*, *H2-T23*, *Tap1*, *Nlrc5)*, *Stat1* and the complement component *C4b*, which we refer to as the hyperinflammatory gene signature in *Nestin*^Cre/+^ *Il12rb2*^fl/fl^ mice, was observed across nearly all cell types (Fig. 4g and Extended Data Fig. 4e). These features were previously referred to as a disease-associated signature in oligodendroyctes^21^ and indicate how decreased neuroprotection in *Nestin*^Cre/+^*Il12rb2*^fl/fl^ mice may foster inflammation.

To test whether the neuroprotective features induced by IL-12 might be crucial for the tissue integrity of the neuroectoderm, we compared our signature to a publicly available dataset from a genome-wide in vivo knockdown screen^22^. Indeed, we noted a decreased CNS essential gene signature corresponding to genes that were found to be relevant for both short-term and long-term survival across oligodendrocytes and neurons in *Nestin*^Cre/+^*Il12rb2*^fl/fl^ mice (Fig. 4h and Supplementary Table 2). Notably, ablation of each of the CNS essential genes led to spontaneous neurodegeneration in mice^22^.

**Extended Data Fig. 5:**
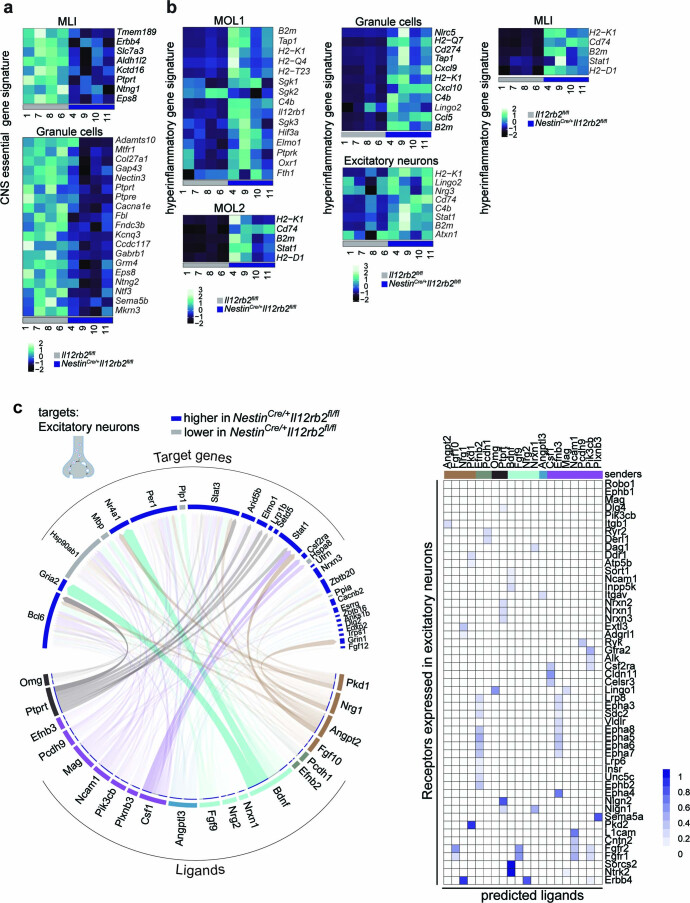
IL-12 induces CNS essential and suppresses hyperinflammatory gene signatures. Nuclei were isolated from the CNS of *Il12rb2*^*fl/fl*^ and *Nestin*^*Cre/+*^*Il12rb2*^*fl/fl*^ male mice (n = 4 per genotype, 8-12 weeks old) at the onset of EAE (10 dpi). (a) and (b) Heatmaps showing the mean gene expression aggregated per mouse for genes of a (a) CNS essential gene signature in MLI and granule cells (b) Hyperinflammatory gene signature in MOL1 and MOL2, Granule cells, excitatory neurons and MLI. (c) Circos plots showing links between unique ligands from senders (ribbon colour indicates subcluster of origin for each ligand; multiple sender cells: ribbon colour black) and predicted associated DE genes in excitatory neurons. Transparency indicates interaction strength, and the ribbon thickness is proportional to the ligand’s regulatory potential. Heatmap (right) displaying potential receptors expressed in excitatory neurons associated with each predicted ligand. Differential gene expression in (a) and (b) was tested using pseudo-bulking and consecutive DEseq2 analysis and is presented in Supplementary Table 3.

To evaluate whether the induction of a neuroprotective gene module and the concomitant suppression of hyperinflammatory features in the presence of IL-12 signaling could be validated across experimental mice, we next performed pseudo-bulk analysis in conjunction with DESeq2 differential expression analysis (Extended Data Fig. 5a,b and Supplementary Table 3). This approach has been reported to reduce false discovery rates (FDRs) and increase external validity in recent benchmarking studies^23^. We confirmed differential expression for 27 genes essential for neuronal survival (gene module: *Erbb4*, *Ntf3*, *Nectin3*, *Ntng2*, *Ptprt*) and function in granule cells and MLIs (Extended Data Fig. 5a), as well as 44 features validating the increased signature of inflammation (gene module: *C4b*, *Hif3a*, *Stat1*, *B2m*, *H2-K1*, *H2-D1*, *Cxcl10*) in *Nestin*^Cre/+^ *Il12rb2*^fl/fl^ mice across all clusters (Extended Data Fig. 5b).

**Extended Data Fig. 6:**
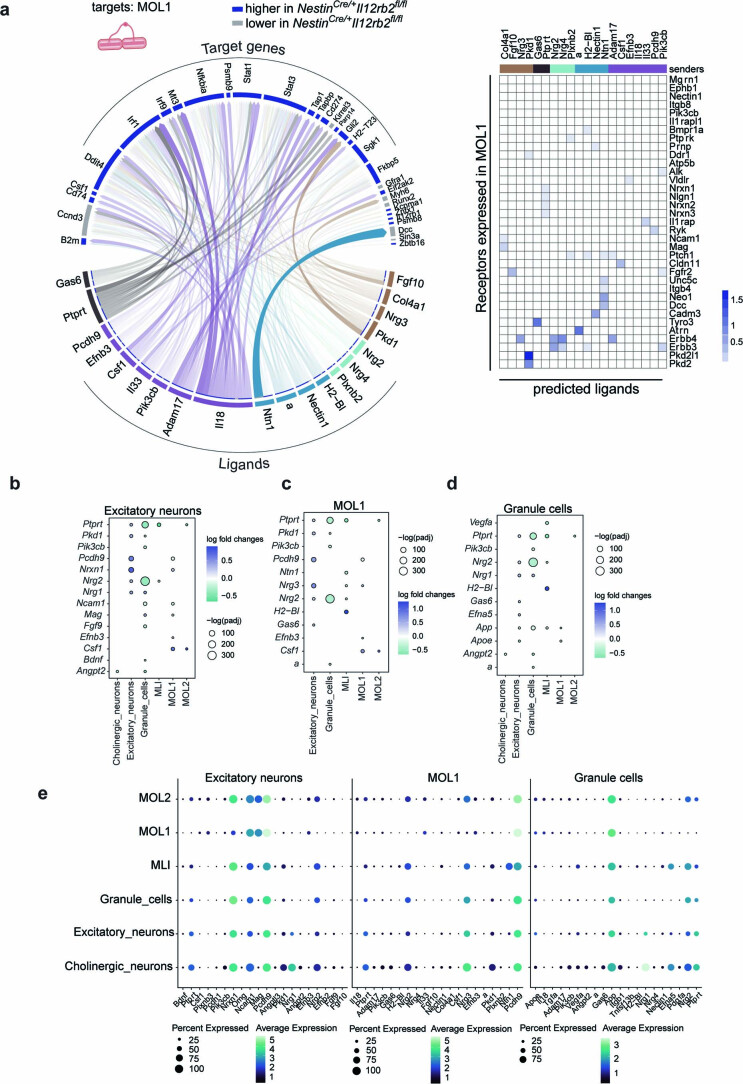
IL-12 sustains trophic factor environment for oligodendrocytes. (a) Circos plots showing links between unique ligands from senders (ribbon colour indicates subcluster of origin for each ligand; multiple sender cells: ribbon colour black) and predicted associated DE genes in MOL1. Transparency indicates interaction strength, and the ribbon thickness is proportional to the ligand’s regulatory potential. Heatmap (right) displaying potential receptors expressed in MOL1 associated with each predicted ligand. (b), (c) and (d) Dot plots showing differential gene expression for differentially expressed ligands in (b) excitatory neurons (c) MOL1 and (d) Granule cells. Dot size indicates significance and color indicates log fold change between *Nestin*^*Cre/+*^*Il12rb2*^*fl/fl*^ and *Il12rb2*^*fl/fl*^ mice in the respective cell types. (e) Dot plots depicting the expression of the predicted ligands (in sender) for the following recipient clusters: excitatory neurons, MOL1 and Granule cells (left to right). Dot size corresponds to the percentage of nuclei expressing the gene in each cluster, and the color represents the average expression level. Differential gene expression in (b), (c) and (d) was tested using a two-sided Wilcoxon rank sum test and applying a Benjamini-Hochberg correction and is presented in Supplementary Table 1.

Neurodegeneration and neuroinflammation can be caused by pathophysiological intercellular communication. To explore how IL-12 affects intercellular communication by IL-12R-expressing neurons and oligodendrocytes, we analyzed ligand–receptor interactions that are altered upon loss of IL-12 using the NicheNet algorithm^24^ (Fig. 4i,j and Extended Data Figs. 5c and 6a–e). We intentionally defined sender cells by their ability to sense and respond to IL-12 (that is, specific neuronal and oligodendrocyte subpopulations), while recipients included all cells captured by snRNA-seq (IL-12R expressing and not expressing). While DEG analysis can only predict direct IL-12-mediated transcriptional changes, this approach allowed us to decipher IL-12-induced, indirect changes. Two common patterns of intercellular communication shaped by IL-12 arose: neuron-intrinsic protection counteracting neurodegeneration and trophic factor release supporting cells of the neuroectoderm (Fig. 4i,j and Extended Data Figs. 5c and 6a–e).

Specifically, NicheNet revealed an IL-12-dependent upregulation of receptor–ligand interactions linked to neuronal survival and development, as well as axonal integrity and growth/regeneration, such as BDNF–NTRK2 (ref. ^25^), PIK3CB–FGFR1/2 and PTPRT–NLGN2 in excitatory neurons (Extended Data Fig. 5c). EFNB3–EPHA4, EFNB2–EPHB6 and EFNB3–EPHB6, which are essential cues for axon guidance, synapse formation and dendritic morphology emerged as dysregulated interactions in the absence of IL-12 (Extended Data Figs. 5c and 6b,e). We noted a similar neuroprotective signature emerging in granule cells, where IL-12 promoted intercellular communication conduits including NRG1–ERBB4, NRG2–ERBB4, EFNA5–EPHA7 and NRXN1–NLGN1, with the latter being implicated in the pathology of MS, neurodegenerative and neuropsychiatric disorders^26^ (Fig. 4j and Extended Data Fig. 6d,e). Additionally, critical pro-survival cues comprising FGF9–FGFR1/FGFR2, FGF10–FGFR1/FGFR2, NCAM1–FGFR1/FGFR2 in excitatory neurons (Extended Data Figs. 5c and 6b,e), as well as VEGFA–NRP2 engagement in granule cells were elicited in response to IL-12 (Extended Data Fig. 6d,e). Several of these top-ranked ligands themselves were DEGs, supporting their contribution to the IL-12-driven tissue-protective neuroimmune cross-talk (Extended Data Fig. 6b–d).

The most pronounced theme observed in the NicheNet analysis of MOL populations (that is, MOL1 and MOL2) was a dysregulated trophic factor environment in the absence of IL-12 receptor signaling (Extended Data Fig. 6a,c,e). Trophic signals shaping the neuron–oligodendrocyte cross-talk that were affected by IL-12 included ADAM17–ERBB4 and neuron-derived NECTIN–FGFR2/FGFR3, NRG2–ERBB3/ERBB4 and FGF10–FGFR2 (Extended Data Fig. 6a). Taken together, our data suggest IL-12 to be critically involved in neuroprotection and shaping the trophic factor milieu within the inflamed CNS. We observed that the dominant source of trophic factors were neurons (Fig. 4j and Extended Data Figs. 5c and 6a,e) highlighting their function as critical homeostatic gatekeepers. Conversely, dysregulation or loss of trophic factor release by neurons in the absence of IL-12 may further propagate the degeneration of neurons, oligodendrocytes and possibly other cell types.

### Neurons mediate IL-12-induced tissue protection in experimental autoimmune encephalomyelitis

**Fig. 5:**
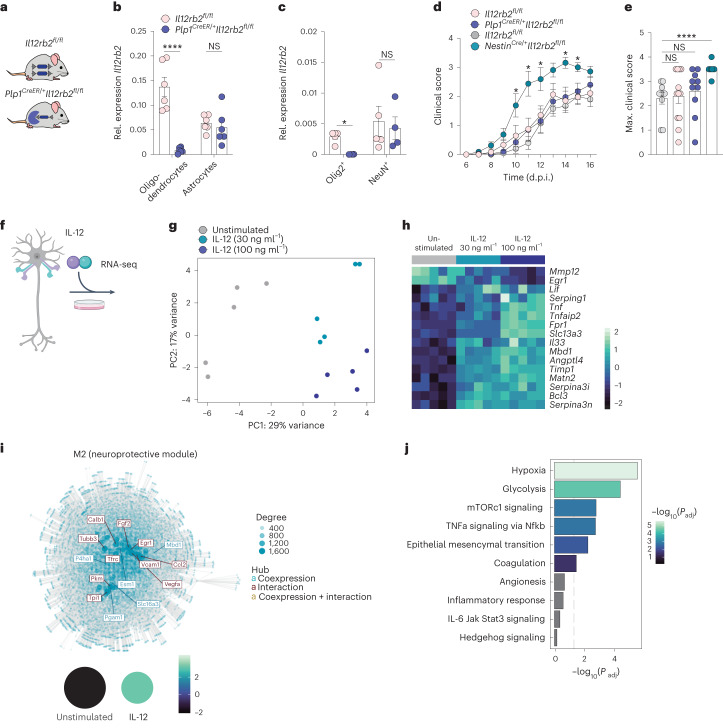
IL-12 engagement in neurons, but not in oligodendrocytes, elicits CNS tissue-protective features during neuroinflammation. **a**, Schematic of *Plp1*^CreER/+^*Il12rb2*^fl/fl^ and *Il12rb2*^fl/fl^ control mice. **b**, *Il12rb2* mRNA expression of CD45^−^CD11b^−^ACSA-2^−^CD140a^−^GalC^+^ oligodendrocytes and CD45^−^CD11b^−^ACSA-2^+^ astrocytes isolated by FACS from the CNS of *Plp1*^CreER/+^ *Il12rb2*^fl/fl^ (*n* = 6, m/f) and littermate controls (*n* = 6, m/f) at 16 d.p.i. Data show the mean ± s.e.m. (data are representative for one of two experiments; unpaired two-tailed *t*-test, ****P ≤ 0.0001; *P* = 0.5967, left to right). **c**, *Il12rb2* mRNA expression of Hoechst^+^Olig2^+^ oligodendrocyte nuclei and Hoechst^+^NeuN^+^ neuronal nuclei isolated by FANS from the CNS of *Plp1*^CreER/+^*Il12rb2*^fl/fl^ (*n* = 4, f) and littermates (*n* = 5; f) at late-stage EAE (16 d.p.i.). Data show the mean ± s.e.m. (data are representative for one of two experiments; two-tailed Mann–Whitney test*,* *P = 0.0159; *P* = 0.9048, left to right). **d**,**e**, Clinical disease course (**d**) and individual maximal EAE scores (**e**) of MOG emulsified in complete Freund’s adjuvant (CFA) induced EAE in *Plp1*^CreER/+^*Il12rb2*^fl/fl^ (*n* = 10; m/f), their littermate controls (*n* = 12, m/f; pink group treated with tamoxifen), *Nestin*^Cre/+^*Il12rb2*^fl/fl^ (*n* = 8, m/f) and littermate *Il12rb2*^fl/fl^ control mice (*n* = 10, m/f; gray group not treated with tamoxifen). Data were pooled from two independent experiments shown as the mean ± s.e.m. Mixed-effects model with Bonferroni post hoc test between *Plp1*^CreER/+^*Il12rb2*^fl/fl^ and *Nestin*^Cre/+^*Il12rb2*^fl/fl^ mice (*P = 0.044; *P = 0.0324; *P = 0.0462; *P = 0.0793; *P = 0.0193; *P = 0.0457; *P* = 0.3357, left to right) in **d** and unpaired two-tailed Mann–Whitney test (*P* = 0.632; *P* = 0.1927; ****P ≤ 0.0001; left to right) in **e**. **f**, Primary neuronal cultures were incubated with IL-12 (30 ng ml^−1^ and 100 ng ml^−1^) for 18 h and subjected to bulk RNAseq (*n* = 5 replicates per group). **g**, PCA of bulk RNAseq data shows a dose-dependent effect of IL-12 on neurons. **h**, Heatmap depicting DEGs (FDR < 0.05) between untreated and IL-12-treated neurons. **i**, Modular differential gene coexpression analysis. Shown is module activity across condition and global gene interaction network of module 2 (neuroprotective module) depicting the most connected genes (hubs). **j**, Overrepresentation analysis depicting the biological components of the neuroprotective module. Overrepresentation was tested using a hypergeometric test and applying a Benjamini–Hochberg correction; m, male; f, female. Source data

**Extended Data Fig. 7:**
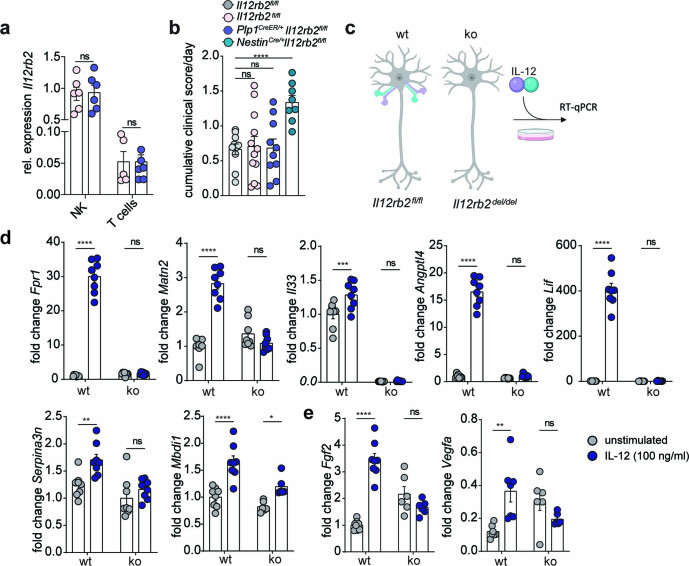
Oligodendrocytes are redundant for the tissue-protective features of IL-12 in EAE. (a) *Il12rb2* mRNA relative expression level in NK cells and CD3^+^ T cells isolated by FACS from the spleens of *Plp1*^*CreER*^*Il12rb2*^*fl/fl*^ (n = 7, m/f) mice and littermate controls (n = 6, m/f) at 16 dpi. Data pooled from two independent experiments and depicted as mean ± s.e.m. (unpaired two –tailed *t*- test, *P* = 0.9941; *P* = 0.8905; left to right). (b) Cumulative clinical scores/day of MOG-CFA induced EAE in *Plp1*^*CreER*^*Il12rb2*^*fl/fl*^ (n = 10, m/f), their littermate controls (n = 12, m/f; pink group treated with tamoxifen), *Nestin*^*Cre/+*^*Il12rb2*^*fl/fl*^ (n = 8, m/f) and littermate *Il12rb2*^*fl/fl*^ control mice (n = 10, m/f; grey group not treated with tamoxifen). Data are pooled from two independent experiments shown as mean ± s.e.m. (unpaired two –tailed *t*- test, *P* = 0.8088; *P* = 0.8927; ****P ≤ 0.0001, left to right). (c) Primary neuronal cultures derived from neonatal *Il12rb2*^*fl/fl*^ (wt) and *Il12rb2*^*del/del*^ (ko) mice (pooled litters) were activated with IL-12 (100 ng/ml) for 18 h and subjected to RT-qPCR analysis. (d) Gene expression analysis of selected DEGs (Fig. 5h) (n = 8 wt and n = 6-8 ko; number of dots represents the number of replicates) and (e) selected genes from module M2 (Fig. 5i) in cultured neonatal neurons (n = 8 wt and n = 6 ko replicates). Gapdh was used as an internal reference gene. All data are normalized to their respective unstimulated controls and are representative for 1 out of 2 experiments. Mean ± s.e.m. Two-way ANOVA with Bonferroni post hoc test (d: ****P ≤ 0.0001, *P* > 0.9999; ****P ≤ 0.0001, *P* = 0.2511; ***P = 0.0006, *P* = 0.9967; ****P ≤ 0.0001, *P* > 0.9999; ****P ≤ 0.0001, *P* > 0.9999; **P = 0.0045, *P* = 0.4946; ****P ≤ 0.0001, *P = 0.0145, left to right and e: ****P ≤ 0.0001, *P* = 0.2033; **P = 0.0016, *P* = 0.2172, left to right; ns= not significant).

To test whether IL-12 signaling in neurons alone was sufficient or whether oligodendrocytes are also needed to sense IL-12 to promote tissue integrity during inflammation, we crossed the *Il12rb2* conditional allele to the *Plp1*^*CreER*^ strain (Fig. 5a) that allowed for the depletion of *Il12rb2*, specifically in oligodendrocytes while sparing neurons (Fig. 5b,c and Extended Data Fig. 7a). In contrast to the *Nestin*^Cre/+^*Il12rb2*^fl/fl^ mice, which lacked *Il12rb2* expression in both populations, *Plp1*^CreER/+^*Il12rb2*^fl/fl^ transgenic mice did not display exacerbated clinical symptoms compared to littermate control mice (Fig. 5d,e and Extended Data Fig. 7b). These data solidify the notion that neurons are the main executors of the IL-12-driven neuroprotective tissue adaptation in EAE.

Our transcriptomic data, however, do not fully exclude the possibility that changes in the neuronal compartment are secondary to the different levels of inflammation observed between *Nestin*^Cre/+^*Il12rb2*^fl/fl^ and *Il12rb2*^fl/fl^ mice, despite displaying similar clinical scores at the onset of disease. Unfortunately, neuroprotective features cannot be tested at steady state due to the absence of MdC infiltration (and therefore IL-12) in the homeostatic CNS (Fig. 3l,m). To overcome these limitations and to solidify the findings of the snRNA-seq dataset, we performed bulk RNA-seq of primary murine neurons stimulated with recombinant IL-12 (Fig. 5f–j). Principal component analysis (PCA) revealed a dose-dependent response to IL-12 in the neuronal transcriptome (Fig. 5g). We identified 1,878 significantly DEGs (*P* ≤ 0.01, log ratio ≥ 0.5; Supplementary Table 4); several of the upregulated genes (1,091 in total) have been linked to promoting neurogenesis (*Fpr1*, *Mbd1*), neuroprotection (*Timp1*, *Angptl4*) and enhancing remyelination (*Timp1*, *Matn2*; Fig. 5h). IL-12 suppressed the expression of *Mmp12*, whose inhibition in neurons attenuates axonal pathology and degeneration^27^ and induced the expression of *Serpina3n*, encoding a granzyme B inhibitor, which prevents direct neuronal killing by T cells and NK cells and induces neuroprotection in EAE^28^ (Fig. 5h). Strikingly, IL-12 triggered the neuronal secretion of trophic factors for oligodendrocytes including those encoded by *Lif and Il33* (Fig. 5h), confirming our in vivo findings. Modular gene coexpression network analysis revealed ten groups of genes (modules) whose expression was jointly altered by IL-12 (Fig. 5i). One of the predicted modules (M2 or neuroprotective module), which was induced by IL-12, harbored gene hubs (that is, genes strongly coexpressed and/or interacting with other genes or both) linked to neurogenesis and neuroprotection (*Fgf2*, *Vegfa*, *Egr1*), axonal regeneration (*Tubb3*), oligodendrocyte precursor cell mobilization (*Ccl2*), endogenous myelin repair (*Vcam1, Fgf2*) and neuronal metabolism (glycolysis (*Tpi1*), copper ion transport (*Slc16a3*); Fig. 5i). Furthermore, IL-12 directly triggered pathways known to exert pro-survival effects in CNS pathology; for instance, hypoxia^29^ and the mTORc1 (ref. ^30^) signaling pathway (Fig. 5j). We performed qPCR for selected genes in neuronal cultures from *Il12rb2*^fl/fl^ versus *Il12rb2*^del/del^ mice (Extended Data Fig. 7c,d) and confirmed that elements of the M2 neuroprotective (that is, *Fgf2*, *Vegfa*) module depended on *Il12rb2* expression in neurons (Extended Data Fig. 7e). Collectively, these results demonstrated a direct neuroprotective and neurotrophic effect of IL-12 in neurons.

## Discussion

The discovery of IL-12 coincided with understanding its powerful effects on NK cells and, only later, T cells as well^31^. IL-12 is firmly linked with type 1 immunity^32^ and is widely held as a major driver of inflammation. However, more than a decade later it became clear that IL-23, which shares the p40 subunit with IL-12, is the pivotal factor for the induction of autoimmune tissue pathologies, including neuroinflammation. IL-23 is required for the formation and maintenance of encephalitogenicity in T_H_ cells^33^.

The observation that IL-12-deficient mice develop stronger tissue inflammation across multiple preclinical models of chronic inflammatory diseases (as reviewed in ref. ^34^) has represented somewhat of a paradox. Cytokines, however, are pleiotropic and specific cytokine receptors are often expressed across different cell types. Cytokine networks involve not only immune cells, but also various cellular entities outside the hematopoietic compartment^35^. A prime example is psoriasiform skin inflammation where IL-12 triggers tissue protection via epithelial cells^9^. Equally, Murphy et al.^36^ demonstrated that germline mutants specifically lacking IL-23 (*Il23a*^−/−^ mice) were completely resistant to joint autoimmune inflammation, whereas the selective absence of IL-12 (*Il12a*^−/−^ mice) led to hyperinflammation and exacerbated bone pathology. In vivo administration of IL-12 in a model of systemic sclerosis alleviated disease pathology, implying immunoregulatory actions on IL-12-responsive fibroblasts^37^. Therefore, one could speculate that the spectrum of IL-12’s actions outside the hematopoietic compartment extends far beyond what we currently know.

**Extended Data Fig. 8:**
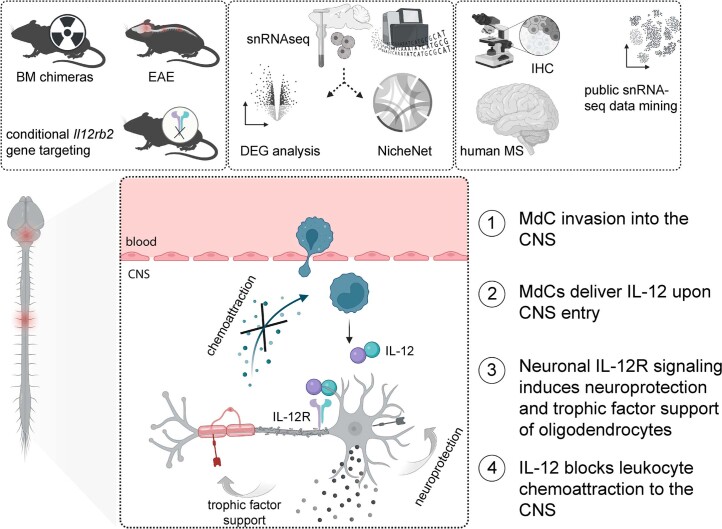
Experimental workflow and working model on IL-12 induced CNS tissue protection during neuroinflammation. Top: We combined bone marrow (BM) chimerism and conditional gene targeting of *Il12rb2* with experimental autoimmune encephalomyelitis (EAE) in mice to unravel the protective role of IL-12 in the CNS neuroectoderm (left). Additionally, we performed snRNAseq coupled with differentially expressed gene (DEG) and NicheNet analysis of the murine CNS at the onset of EAE which revealed the underlying mechanisms of IL-12 induced tissue protection in neurons (middle). We confirmed similar expression of the IL-12R machinery in human MS patients by IHC and snRNAseq data mining (right), suggesting similar mechanisms apply in mice and humans. Bottom: Monocyte derived cells (MdCs) invade the CNS during neuroinflammation and produce IL-12 upon entry. IL-12 can be sensed by neuronal cells which react by (a) intrinsic neuroprotection, (b) trophic factor support to oligodendrocytes and (c) blocking chemoattraction of other leukocytes.

Here, we identified two CNS-endogenous neuroectodermal IL-12 sensors: neurons and oligodendrocytes. Whereas conditional deletion of *Il12rb2* in immune cells was dispensable for IL-12-mediated tissue protection, the loss of IL-12 sensing in neuroectodermal cells, and more specifically, in neurons, led to hyperinflammation during EAE, phenocopying the *Il12rb2* germline mutation that has been reported before^5^. The tissue pathology of *Nestin*^Cre/+^*Il12rb2*^fl/fl^ mice was characterized by severe CNS tissue damage coupled with markedly increased inflammatory infiltrates, which might be a consequence of increased tissue damage^38^. Alternatively, IL-12 might directly interfere with leukocyte attraction through neurons as we for instance detected increased *Ccl2* expression in neurons of *Nestin*^Cre/+^*Il12rb2*^fl/fl^ mice. In fact, the concept of the neuroectoderm as an active immunomodulator, rather than passive bystander of inflammation, has emerged in diverse forms of T cell-mediated autoimmune brain pathologies^39,40^. Neurons, in particular, can influence CNS immune responses in both protective^41^ and detrimental^42^ ways. In addition to regulating immune responses, neurons have been demonstrated to be key players in counteracting CNS tissue damage via trophic factor release in the context of diverse brain ailments^43^. We found that IL-12 induced transcriptional adaptation of the neuroectoderm and specifically instructed neuroprotective cues and trophic factor support by neuronal cell populations within the inflamed CNS (Extended Data Fig. 8). Members of the fibroblast growth factor (FGF) family, for instance, were consistently upregulated by IL-12 and have been suggested to stimulate remyelination and enhance neurogenesis^44^ among its broad repertoire of CNS-protective functions^45^.

The loss of IL-12, was accompanied by a global hyperinflammatory transcriptomic signature in both neurons and oligodendrocytes, including genes linked to antigen presentation (for example, *B2m*) and type 1 signature (for example, *Stat1*) indicating pathogenic processes that further potentiate inflammation^39^. Neuronal MHC class I expression mediates a broad repertoire of functions in the steady-state CNS^46^, while the upregulation of MHC class I and Stat1 renders neurons prone to CD8^+^ cytotoxic T cell-mediated degeneration^42^ and impedes CNS repair^47^, suggesting increased neuronal vulnerability in the absence of IL-12. Conversely, a recent report suggested a detrimental role of IL-12 in Alzheimer’s disease-like disease pathology^12^. The opposing effects in acute neuroinflammation versus chronic neurodegeneration are best explained by differences in the pathophysiology of the respective model. These include the cytokine context (inflammatory milieu including IL-23 and GM-CSF in EAE, which is absent in Alzheimer’s disease-like APP/PS1 mice^35^), the sources and amount of IL-12 (high in MdCs in EAE and lower in glial cells in APP/PS1 mice), the nature of tissue damage (multifocal disseminated in EAE and more restricted to the cerebrum in APP/PS1 mice) as well as CNS region-specific transcriptomic changes of neuroectodermal cells. IL-12, therefore, joins the ranks of other molecules with opposing functions in inflammation versus degeneration of the CNS (for example, GM-CSF^48,49^).

A neuroprotective effect of IL-12 on neurons, uncoupled from the effect of inflammation, however, has also been reported in diverse in vivo and in vitro settings. For instance, administration of IL-12 after EAE induction ameliorates the disease outcome in mice^50,51^. Similarly, IL-12 was shown to exert a therapeutic effect in an in vivo model of spinal cord injury. Local administration of IL-12 into the injured sites induced the expression of neurotrophic factors (BDNF, NT-3) in CNS lesions, thereby enhancing endogenous axonal regeneration and remyelination^52^. IL-12 also promoted regeneration in primary mouse superior cervical ganglion cell cultures, where IL-12 enhanced neurite outgrowth^53^.

In summary, our study sheds light on a 20-year-old conundrum by elucidating the cellular targets and molecular underpinnings of a neuroimmune cross-talk orchestrated by IL-12 in neuroinflammation (Extended Data Fig. 8). The observation that the IL-12 receptor is expressed in both mouse and human neuroectoderm raises the possibility that IL-12 might trigger a similar reparative mechanism also in the human CNS. Similar to the findings in mice, we found that cortical GM excitatory neurons—healthy, but also underlying meningeal inflammation in MS—expressed *IL12RB2*, possibly suggesting a state of readiness to sense IL-12. Thereby, IL-12 sensing during disease onset might counteract early neurodegenerative pathways as recently described in human MS^54^. The neuroprotective function of IL-12 might contribute to an inflammation-induced emergency break crucial to prevent immunopathology. We hypothesize that such a mechanism applies not only in autoimmunity but also in other inflammatory CNS diseases, for instance, during pathogen-induced meningitis or encephalitis. The fact that IL-12 confers neuroprotection may provide a new avenue of disease intervention in both neuroinflammatory and degenerative diseases.

## Methods

### Mice

All procedures were reviewed and approved by the Swiss Veterinary Office and performed according to institutional and federal guidelines. All transgenic mouse strains were bred on the C57BL/6 background (The Jackson Laboratory, 000664) and were maintained under optimized hygienic conditions (22 °C; 45–65% humidity; 12-h light cycle); standard laboratory diet and water were freely available. B6.C-Tg(CMV-Cre)1Cgn/J^55^ (006054) were crossed to *Il12rb2*^fl/fl^ (ref. ^9^) mice to generate *Il12rb2*^del/del^ full knockout mice. B6.Cg-Tg(Nes-cre)1Kln/J^56^ (003771), *B6.Cg-Tg(Vav1-icre)iKi*^57^ (008610) and *B6.Cg-Tg(Cd4-cre)1Cwi/BfluJ*^58^ (017336) were originally purchased at The Jackson Laboratory. *Ncr1*^Cre^ mice were kindly provided by E. Vivier. C57BL/6 wild-type mice were purchased from Janvier Labs (Le-Genest Saint-Isle, France).

Age-matched, sex-matched (male and female) and litter-matched 8- to 12-week-old mice were used for all in vivo experiments. For primary neuronal and oligodendrocyte cultures, pups were euthanized between postnatal day 0 (P0) and 4 (P4). For the sample size calculation of mouse experiments, we used an alpha = 0.05 and a power of 0.8 (no blocking for age, sex and litter in the power calculation). We used data from previous experiments for the mean and standard deviation to calculate the standardized effect size using G Power. We determined the required sample size to be able to see a 30% difference in the cumulative score (area under the curve) for disease course and 70% by qPCR/gene expression for assessing the targeting efficacy/specificity of our strains.

### Human samples

Brain tissue from three individuals with MS was obtained from the archives of the Institute of Neuropathology at the University Hospital Zurich, Switzerland. Informed consent for autopsy was given by the next of kin in all cases. Case series do not need institutional review board approval according to Swiss legislation.

### Experimental autoimmune encephalomyelitis induction

EAE was induced by injection of 200 μg of MOG epitope MOG_35-55_ (RP10245, Genscript) emulsified in complete Freund’s adjuvant (CFA; 231131, BD). All mice received two subcutaneous injections into the flank and a single intraperitoneal injection of 50–100 ng pertussis toxin (PT; List Biological Laboratories, 179A). The mice received a second injection of PT on 2 d.p.i. Of note, by reducing the concentration of PT, *Il12rb2*^fl/fl^ mice developed a mild disease, whereas *Il12rb2*^del/del^ mice were still hypersusceptible, but within animal welfare guidelines. EAE clinical scores were defined as follows: no detectable signs of EAE, 0; tail limp at distal end, 0.5; entirely limp tail, 1; limp tail and hind limb weakness (occasional grid test positive), 1.5; unilateral partial hind limb paralysis (one leg constantly falls through the grid), 2; bilateral partial hind limb paralysis, 2.5; complete bilateral hind limb paralysis, 3; complete bilateral hind limb paralysis and partial forelimb paralysis, 3.5; complete paralysis of fore and hind limbs (moribund), 4. In case the clinical symptoms couldn’t be assigned to one or the other score, we used in-between scoring intervals of 0.25, which allowed a more accurate description of the clinical symptoms. Withdrawal criteria included clinical score ≥ 3 for more than 7 d, clinical score ≥ 3.5 for more than 3 d, weight loss > 25%, as well as pain symptoms.

### Tamoxifen treatment

Tamoxifen (Sigma) was dissolved in ethanol and corn oil to 25 mg ml^−1^ and administered in 200-μl doses via oral gavage (5 mg per dose).

### Generation of bone marrow chimeras

Host animals were lethally irradiated with a split dose of 2 × 550 rad at a 24-h interval before receiving an intravenous injection of 5 × 10^6^ donor bone marrow cells. The immune reconstitution period before EAE induction was 6 weeks. Transplantation of *Il12rb2*-null (*Il12rb2*^del^) bone marrow into wild-type (*Il12rb2*^fl^) mice was used to assess the effect of *Il12rb2* depletion in hematopoietic cells (that is, peripheral and CNS-invading leukocytes). Transfer of wild-type (*Il12rb2*^fl^) bone marrow into *Il12rb2*-null (*Il12rb2*^del^) mice was used to assess the effect of IL-12 sensing in radioresistant and non-hematopoietic (CNS resident) cell types, that is, neuroectoderm, tissue-resident microglia and tissue-resident memory T (T_RM_) cells. Wild-type (*Il12rb2*^fl^) into wild-type (*Il12rb2*^fl^) and *Il12rb2*-null (*Il12rb2*^del^) mice into *Il12rb2*-null (*Il12rb2*^del^) mice served as positive and negative controls, respectively (Table 1).

### Natural killer cell depletion

To deplete NK cells in vivo, C57BL/6 mice were injected intraperitoneally with 200 μg of anti-NK1.1 monoclonal antibody (BP0036, clone PK136, BioXcell) at days 0 and 2 after EAE induction, and after every 6 d. The percentage of NK cell depletion was assessed in the CNS and spleen.

### Cell isolation from the adult mouse CNS

Mice were euthanized through CO_2_ asphyxiation and transcardially perfused with ice-cold PBS.

For leukocyte isolation, whole brain and spinal cord was harvested, cut into small pieces and digested with 0.4 mg ml^−1^ Collagenase IV (9001-12-1, Sigma-Aldrich) and 0.2 mg ml^−1^ deoxyribonuclease I (DNase I; E1010, Luzerna) in HBSS (with Ca^2+^ and Mg^2+^; 14025-050, Gibco) for 40 min at 37 °C. The digested tissue was then mechanically dissociated using a 19-gauge needle and filtered through a 100-μm cell strainer (800100, Bioswisstec). CNS single-cell suspensions were further enriched by 30% Percoll (P4937, GE Healthcare) gradient centrifugation (1,590*g*, 30 min, at 4 °C, with no brakes).

To enrich for and improve oligodendrocyte viability for downstream FACS sorting and RNA isolation, CNS tissue was subjected to enzymatic digestion using papain (LS003126, Worthington) for 30 min at 37 °C and the enzymatic reaction stopped with ovomucoid trypsin inhibitor (LS003086, Worthington). Myelin debris was again removed by 30% Percoll (P4937, GE Healthcare) gradient centrifugation.

### Flow cytometry

Before surface labeling, cells were incubated with purified anti-mouse CD16/CD32 (clone 93, BioLegend) for 10 min on ice to prevent nonspecific binding of primary antibodies. Single-cell suspensions were then directly incubated with the primary surface antibody cocktail in PBS for 20 min at 4 °C. Following a wash step with PBS (350*g*, at 4 °C) the cells were then incubated in the secondary surface antibody cocktail (fluorochrome-conjugated streptavidin) for 20 min at 4 °C. For intranuclear labeling with Ki67 and Foxp3, cells were fixed and permeabilized utilizing the Foxp3/Transcription Factor Staining Buffer Set (eBioscience) according to the manufacturer’s instructions and subsequently incubated in the intranuclear antibody mixture overnight at 4 °C. Anti-mouse fluorochrome-conjugated monoclonal antibodies used in this study were: CD8 (564920, BD Biosciences, clone 53-6.7, BUV805, dilution 1:100), CD274 (124311, BioLegend, clone 10F.9G2, APC, dilution 1:200), CD274 (25-5982-82, eBioscience, clone MIH5, PE-Cy7, dilution 1:500), CD45R (552094, BD Biosciences, clone RA3-6B2, APC-Cy7, dilution 1:200), CD38 (102714, BioLegend, clone 90, AF488, dilution 1:300), granzyme B (515406, BioLegend, clone GB11, AF647, dilution 1:100), I-A/I-E (107622, BioLegend, clone M5/114.15.2, AF700, dilution 1:400), Siglec-F (564514, BD Biosciences, clone E50-2440, BB515, dilution 1:400), NK1.1 (566502, BD Biosciences, clone PK136, BB700, dilution 1:100), CD45 (564279, BD Biosciences, clone 30-F11, BUV395, dilution 1:200), CD4 (564667, BD Biosciences, clone GK1.5, BUV496, dilution 1:200), Ly6G (565707, BD Biosciences, clone 1A8, BUV563, dilution 1:100), CD19 (565076, BD Biosciences, clone 1D3, BUV661, dilution 1:200), CD44 (612799, BD Biosciences, clone IM7, BUV737, dilution 1:200), CD64 (139309, BioLegend, clone X54-5/7.1, BV421, dilution 1:100), Ki67 (566109, BD Biosciences, clone B56, BV480, dilution 1:100), Ki67 (652420, BioLegend, clone 16A8, AF700, dilution 1:200), F4/80 (MCA497A488, AbD Serotec, clone BM8, BV510, dilution 1:50), F4/80 (MCA497A488, AbD Serotecclone, clone CI:A3-1, AF647, dilution 1:200), CD62L (104433, BioLegend, clone MEL-14, BV570, dilution 1:100), CX3CR1 (149027, BioLegend, clone SA011F11, BV605, dilution 1:400), CD11b (101239, BioLegend, clone M1/70, BV650 dilution 1:400), Ly-6C (128037, BioLegend, clone HK1.4, BV711, dilution 1:400), MerTK (78-5751-82, eBioscience, clone DS5MMER, SuperBright780, dilution 1:50), CD103 (121406, BioLegend, clone 2E7, PE, dilution 1:100), TCR beta chain (109209, BioLegend, clone H57-597, PE-Cy5, dilution 1:400), CD11c (35-0114-82, eBioscience, clone N418, PE-Cy5.5, dilution 1:400), CD49d (103618, BioLegend, clone R1-2, PE-Cy7, dilution 1:100), Foxp3 (61-5773-82, eBioscience, clone FJK-16s, PE-eFlour610, dilution 1:200), CD90.2 (105324, BioLegend, clone 30-H12, Pacific Blue, dilution 1:200), TCRγδ (46-5711-82, eBioscience, clone GL3, PerCP-eFlour710, dilution 1:100), CD88 (135811, BioLegend, clone 20/70, Biotin, dilution 1:200), Arginase-1 (17-3697-82, Invitrogen, clone A1exF5, APC, dilution 1:200), IL-1 beta Pro-form (12-7114-82, eBioscience, clone NJTEN3, PE, dilution 1:200), CD14 (64-0141-82, Invitrogen, clone Sa2-B, SuperBright 645, dilution 1:200), CD49b (108918, BioLegend, clone DX5, Pacific Blue, dilution 1:200), streptavidin (564923, BD Biosciences, BUV805-conjugated, dilution 1:200), NKp46 (46-3351-82, eBioscience, clone 29A1.4 PerCP-eFlour710, dilution 1:200), NK1.1 (108749, BioLegend, clone PK136, BV785, dilution 1:200), Ly-6C (553104, BD Biosciences, clone AL-21, FITC, dilution 1:400), CD11c (117308, BioLegend, clone N418, PE, dilution 1:200), CD45R (562290, BD Biosciences, clone RA3-6B2, PE-CF594, dilution 1:400) and F4/80 (123112, BioLegend, clone BM8, PE-Cy5, dilution 1:400).

Sample data were acquired on a 5-laser Aurora spectral analyzer (Cytek Biosciences; SpectroFlo v2 Software), FACS Symphony A5 (BD Biosciences; FACS Diva Software v9.1) or LSR II Fortessa (BD Biosciences; FACS Diva Software v9). Flow cytometry data were analyzed using FlowJo (version 10.8.0, Tree Star) and Rstudio (version 4.0.1). Doublet discrimination was performed based on SSC-A/H, FSC-A/H and dead cell exclusion by using a Fixable Viability Kit (Near-IR staining, dilution 1:1,000, BioLegend).

### Phosphorylation of STAT and pSTAT4 flow cytometry

Spleens from untreated healthy mice were pushed through a 70-μm filter and washed once with 1× PBS (350*g*, 10 min, at 4 °C). Erythrocytes were removed by incubation with RBC lysis buffer (8.3 mg ml^−1^ ammonium chloride, 1.1 mg ml^−1^ potassium bicarbonate and 0.37 mg ml^−1^ EDTA) for 5 min on ice. After lysis, samples were washed once in 1× PBS, re-filtered and seeded in 24-well plates (500,000 cells per well) in RPMI medium (supplemented with penicillin–streptomycin, 10% FCS, l-glutamine). Cells were cultured with anti-CD3 (5 μg ml^−1^; 3C11; BioXCell) and anti-CD28 (5 μg ml^−1^; 37.51; BioLegend) for 48–72 h at 37 °C, 5% CO_2_. Cells were then washed in 1× PBS (350*g*, 10 min, at 4 °C) and stained with a fixable viability dye (Zombie NIR Fixable Viability Kit, BioLegend) in PBS for 20 min at 4 °C. Following cell viability staining, the cells were resuspended in pre-warmed (37 °C) recombinant IL-12-containing (20 ng ml^−1^; PeproTech) medium for 15 min to induce phosphorylation of STAT4. The samples were fixed by directly adding 4% paraformaldehyde (PFA) solution (pH 7.4) to a final concentration of 2% (10 min, room temperature (RT)). Cells were centrifuged and immediately resuspended in 1 ml of ice-cold methanol for permeabilization (30 min on ice). Subsequently, cells were washed twice with FACS buffer (1% BSA in PBS). Five microliters of anti-mouse pSTAT4 (12-9044-42, eBioscience, clone 4LURPIE, PE) was added to the cell pellet right before the addition of the surface antibody cocktail. Cells were incubated for 30 min at 4 °C, washed and resuspended in 200 μl of FACS buffer for data acquisition.

### Fluorescence-activated cell sorting

Cell sorting was performed using a BD FACS Aria III (FACSDIVA Software v9). Anti-mouse fluorochrome-conjugated monoclonal antibodies used in this study were: CD45 (103126, BioLegend, clone 30-F11, Pacific Blue, dilution 1:600), CD11b (101263, BioLegend, clone M1/70, BV510, dilution 1:400), CD44 (103049, BioLegend, clone IM7, BV650, dilution 1:400), CX3CR1 (149027, BioLegend, clone SA011F11, BV605, dilution 1:400), Ly-6C (128037, BioLegend, clone HK1.4, BV711, dilution 1:400), LY6G (127606, BioLegend, clone 1A8, FITC, dilution 1:400), NK1.1 (25-5941-82, eBioscience, clone PK136, PE-Cy7, dilution 1:400), O4 (130-117-711, Miltenyi, clone REA576, PE, dilution 1:25), ACSA-2 (130-116-245, Miltenyi, REA969, APC, dilution 1:25), CD88 (135811, BioLegend, clone 20/70, Biotin, dilution 1:200), F4/80 (MCA497A488, AbD Serotec, clone CI:A3-1, AF647, dilution 1:200), CD140a (135911, BioLegend, clone APA5, PE-Cy7, dilution 1:400), TER-119 (17-5921-81, BioLegend, clone Ter-119, APC, dilution 1:200), CD3 (100236, BioLegend, clone 17A2, APC, dilution 1:200), CD31 (102507, BioLegend, clone MEC13.3, PE, dilution 1:200), CD31 (102427, BioLegend, clone 390, BV605, dilution 1:400), CD4 (100548, BioLegend, RM4-5, BV605, dilution 1:400), streptavidin (557598, BD Biosciences, PE-Cy7-conjugated, dilution 1:400) and GalC (FCMAB312F, Milli-Mark, FITC, clone mGalC, dilution 1:10).

Doublet exclusion was performed based on SSC-A/H, FSC-A/H and dead cell exclusion by using a Fixable Viability Kit (Near-IR staining, dilution 1:500, BioLegend).

### Quantitative RT–PCR

Total RNA was isolated from sorted cells and primary cultures using either the RNeasy Micro Kit (74004, Qiagen) or the QuickRNA Microprep Kit (R1051, Zymo Research) according to the manufacturer’s instructions. Complementary DNA (cDNA) was synthesized using the M-MLV Reverse Transcriptase (28025013, Invitrogen) and qRT–PCR was performed on a CFX384 Touch Real-Time PCR Detection System (Bio-Rad) using SYBR Green (Bio-Rad). Total RNA was isolated from sorted Hoechst^+^Olig2^+^ and Hoechst^+^NeuN^+^ nuclei using the RNeasy Micro Kit (74004, Qiagen). Nuclear RNA was reverse transcribed to cDNA with the RevertAid H Minus First Strand cDNA Synthesis Kit (K1631, Thermo Fisher Scientific) according to the manufacturer’s instructions. Gene expression was calculated as 2^−^^ΔCt^ relative to *Pol2* as the endogenous control.

Primers for *Il12rb2:*

5′-CTGCGAGATCTGAGACCGT-3′; (forward)

5′-AACAGGCTCTTCCTCTGGTGT-3′ (reverse)

Primers for *Il12a:*

5′-TACTAGAGAGACTTCTTCCACAACAAGAG-3′; (forward)

5′-TCTGGTACATCTTCAAGTCCTCATAGA-3′ (reverse)

Primers for *Il12b:*

5′-GACCATCACTGTCAAAGAGTTTCTAGAT-3′; (forward)

5′-AGGAAAGTCTTGTTTTTGAAATTTTTTAA-3′ (reverse)

Primers for *Il12rb1:*

5′-GGACCAGCAAACACATCACCTT-3′; (forward)

5′-GTGATGGCTGCTGCGTTG-3′ (reverse)

Primers for *Pol2:*

5′-CTG GTC CTT CGA ATC CGC ATC-3′; (forward)

5′-GCT CGA TAC CCT GCA GGG TCA-3′ (reverse)

Gene expression analysis of neuronal cultures was performed using the TaqMan Fast Advanced Master Mix (4444557, Thermo Fisher Scientific) and TaqMan primers for *Fgf2* (Mm01285715_m1, Thermo Fisher Scientific), *Vegfa* (Mm00437306_m1, Thermo Fisher Scientific), *Serpina3n* (Mm00776439_m1, Thermo Fisher Scientific), *Mbd1* (Mm00522100_m1, Thermo Fisher Scientific), *Il33* (Mm00505399_m1, Thermo Fisher Scientific), *Matn2* (Mm01166023_m1, Thermo Fisher Scientific), *Lif* (Mm00434761_m1, Thermo Fisher Scientific), *Angptl4* (Mm00480431_m1, Thermo Fisher Scientific) and *Fpr1* (Mm00442803_s1, Thermo Fisher Scientific).

### Primary neuronal cultures

Dissociated cerebellar cells were prepared and maintained as previously described^59^. Briefly, cerebellar cells from P0–P4 mouse pups (C57BL/6) were dissociated and plated on poly-l-lysine-coated, 12-well plates (90,000 cells per well). Cells were maintained in Neurobasal Plus medium supplemented with B27 Plus (A3653401, Thermo Fisher Scientific) and 2 mM GlutaMax (35050061, Thermo Fisher Scientific).

### Primary oligodendrocyte cultures

For murine primary oligodendrocytes cultures, brains were collected from P2–P5 pups and dissociated using the Neural Tissue Dissociation Kit (P) (Miltenyi Biotec, 130-092-628) on a gentleMACS Octo Dissociator (Miltenyi Biotec, 130-096-427). The resulting single-cell suspension was labeled with O4-conjugated magnetic MicroBeads (Miltenyi Biotec, 130-096-670) and passed through LS columns (Miltenyi Biotec, 130-042-401) to positively select for oligodendrocytes. After isolation, the primary oligodendrocytes were plated at a density of 100,000 cells on plates (Corning, 3524) coated with 10% PLL (Sigma, P4832-50ML) and 1.5% laminin (Sigma, L2020) in 500 μl growth medium (MACS Neuro Medium, 130-093-570) containing 2% MACS Neuro Brew-21 (130-093-566), 1% penicillin–streptomycin (Gibco, 15070063) and 0.5 mM L-glutamine (25030081), 10 ng ml^−1^ human PDGF-AA (130-093-977) and 10 ng ml^−1^ human FGF-2 (130-093-837). At days 2 and 4 of culture, 250 µl of medium was removed and an additional 250 μl of fresh growth medium was added. On day four, cells were stimulated as indicated with 100 ng ml^−1^ of IL-12p70 (PeproTech, 210-12) and harvested for western blot analysis.

### Western blot

Primary neuronal cells at 14 days in vitro (DIV 14) or oligodendrocytes at DIV 4 were stimulated with 100 ng ml^−1^ of recombinant murine IL-12p70 (210-12, PeproTech) for 5 min, 10 min or 15 min. After stimulation, neurons and oligodendrocytes were harvested and lysed in EBC buffer (50 mM Tris (pH 8.0), 120 mM NaCl, 0.5% Nonidet P-40) containing complete miniprotease inhibitor (11836153001, Roche Diagnostics) and phosphatase inhibitor mixture 1 and 2 (P2850 and P0044, Sigma-Aldrich) for 20 min at 4 °C. The clear cell lysate was collected by centrifugation (10,000*g*, 4 °C, 20 min) and supernatants were stored at −80 °C. Samples were run on Tris-glycine polyacrylamide gels and proteins were transferred to PVDF membranes. Primary antibodies (Cell Signaling: rabbit anti-mouse Stat4, 2653, dilution 1:2,000; rabbit anti-mouse Phospho-Stat4-Tyr693, 4134, dilution 1:2,000; mouse monoclonal anti-actin, MAB1501, Merck, dilution 1:5,000) were incubated with the membranes in Tris-buffered saline (TBS) with 0.05% Tween 20 (TBST), including 5% Western Blocking Reagent Solution (11921673001, Roche) overnight at 4 °C. Membranes were washed 5× for 5 min in TBST before the addition of secondary antibodies.

Horseradish peroxidase (HRP)-coupled goat anti-rabbit secondary antibodies (ab6721, Abcam; dilution 1:20,000) and donkey anti-mouse secondary antibodies (715-625-151, Jackson ImmunoResearch, Alexa Fluor 680; dilution 1:10,000) were incubated for 30 min at RT, and membranes were washed again five times for 5 min in TBST. Fluorescence signals were captured using the Odyssey CLx Imager. SuperSignal West Pico Chemiluminescent Substrate (34579, Thermo Fisher Scientific) was applied to visualize HRP-labeled antibodies and developed using the Fujifilm Luminescent Image Analyzer LAS-1000 plus & Intelligent Dark Box II (Fujifilm). Western blot membranes were re-probed for pStat4 and Stat4 using a mild stripping protocol from Abcam: briefly, membranes were incubated for 5–10 min with mild stripping buffer (200 mM glycine, 20 mM SDS, 0.01% Tween 20, pH 2.2) followed by 10 min incubation with PBS and 5 min incubation with TBST. This process was repeated twice. Images were processed and analyzed using ImageJ version 1.53t^60^.

### Multiplex RNA fluorescence in situ hybridization

Frozen brain tissue was placed in a tissue mold (SA62534-15, Sakura) and submerged in Tissue-Tek freezing medium (4583, Sakura). Then, 10-µm-thick brain tissue sagittal sections were cut using a cryostat (Thermo Fisher Scientific, HM 560), mounted on SuperFrost Plus slides (500621, R. Langenbrink) and dried for 1 h at −20 °C. Tissue processing for RNAscope multiplex staining was carried out following the manufacturer’s protocol for fresh-frozen sections. Briefly, tissue was fixed in freshly prepared 4% PFA (pH 7.4) for 30 min at 4 °C, followed by alcohol dehydration and exposure to H_2_O_2_ for 10 min then Protease IV (322340, Bio-Techne) for 30 min, both at RT. Sections were then incubated for 2 h with target probes at 40 °C in a HybEZ Hybridization System (321711, Bio-Techne). The following RNAscope target probes were used: Mm-Il12rb1 (488761, Bio-Techne), Mm-Il12rb2 (451301, Bio-Techne), Mm-Aldh1l1-C2 (405891, Bio-Techne), Mm-Slc1a3-C2 (430781, Bio-Techne), Mm-Gfap-C2 (313211, Bio-Techne), Mm-Sox10-C2 (435931, Bio-Techne), Mm-Tmem119-C3 (472901, Bio-Techne), Mm-Sall1-C3 (469661, Bio-Techne), Mm-Rbfox3-C3 (313311, Bio-Techne) and Mm-Map2-C3 (431151, Bio-Techne). Signal amplification was achieved using the RNAscope Multiplex Fluorescent Kit v2 (323110, Bio-Techne), following the manufacturer’s protocol. Probes were labeled with Opal 520 (dilution 1:500, C2 probe, FP1487001KT, Perkin Elmer), Opal 570 (dilution 1:500, C1 probe, FP1488001KT, Perkin Elmer) and Opal 690 (dilution 1:500, C3 probe, FP1497001KT, Perkin Elmer) and three-dimensional image stacks (1-µm step size, ×40 objective) of stained sections were acquired on a Leica TCS SP5 confocal laser scanning microscope using a HCX PL APO lambda blue ×63 oil ultraviolet objective controlled by LAS AF scan software v4.0 (Leica Microsystems).

### CNS tissue homogenization and protein extraction

Murine brain and spinal cord were isolated as described above and snap-frozen in liquid nitrogen for storage at −80 °C. For protein extraction, samples were subjected to mechanical homogenization in Cell Lysate Buffer [(1 mM EGTA, 1 mM EDTA, 1% Triton X-100, TBS buffer pH 7.5 (20 mM Tris, 150 mM NaCl)]. All buffers were kept on ice and the final Cell Lysate Buffer was supplemented with cOmplete Mini EDTA-free Protease Inhibitor Cocktail (11836170001, Roche, one tablet per 25 ml) and PhosSTOP (4906845001, Roche, one tablet per 10 ml) immediately before use. The initial homogenization was performed mechanically (two metal beads per sample) using a TissueLyserLT (85600, Qiagen) homogenizer (50 oscillations per second for 10 min), followed by subsequent incubation on ice for 30 min and centrifugation (full speed, 10 min, 4 °C). Protein concentrations of each sample were determined using the Pierce BCA Protein Assay Kit (23225, Thermo Fisher Scientific) according to the manufacturer’s protocol using the Photometer Tecan Infinite 200 M (Tecan).

### ELISA analysis

Enzyme-linked immunosorbent assay (ELISA) to detect IL-12/IL-23 total p40 (DY499, R&D) was performed according to the manufacturer’s instructions. All samples were analyzed in duplicate. Absorption was read at 450 nm and 570 nm (for wavelength correction) on a microplate reader (Infinite 200M, Tecan) and analyzed using the Magellan Software (Tecan).

### Immunofluorescence and imaging of murine brain tissue

Mice were euthanized through CO_2_ asphyxiation and transcardially perfused with ice-cold PBS. Brains were carefully removed, fixed in 4% PFA (11762.00500, Morphisto) for 24 h at 4 °C, rinsed in PBS and then incubated in 30% sucrose in PBS at 4 °C for 24–72 h. Each brain hemisphere was embedded in Cryo Embedding Medium (41-3011-00, Medite) and frozen on dry ice. Free-floating brain sections were cut at a thickness of 35–45 μm using a Hyrax C60 cryostat (Zeiss). Sections were washed three times with PBS and incubated in 10% normal goat serum (PCN5000, Thermo Fisher Scientific) and 0.5% Triton X-100 (Sigma-Aldrich, T8787-100ML) in PBS (2 h, RT) for blocking and permeabilization. Sections that were labeled with antibodies derived from mouse were additionally blocked for another 45 min at RT with the M.O.M. Mouse Ig Blocking Reagent (90 µl in 2.5 ml PBS; BMK-2202, Vector Laboratories). Sections were incubated with the primary antibody cocktail overnight at 4 °C in 5% normal goat serum and 0.5% Triton X-100 in PBS.

The following primary antibodies were used: rat anti-GFAP (13-0300, Thermo Fisher Scientific; dilution 1:400), rabbit anti-Calbindin (ab108404, Abcam, clone EP3478; dilution 1:500), mouse anti-NeuN (MAB377, Chemikon, clone A60; dilution 1:500), mouse CC1 Anti-APC (Ab-7; OP80-100UG, Merck; dilution 1:200) and mouse anti-Olig2 (66513-1-IG, Thermo Fisher Scientific; dilution 1:200). After washing, sections were incubated with the respective fluorochrome-conjugated secondary antibodies (for example, goat anti-mouse/anti-rabbit/anti-rat and streptavidin, conjugated to Alexa Fluor 488, 594, 633 and 647, all purchased from Thermo Fisher Scientific; dilution 1:500) for 2 h at RT in 5% normal goat serum and 0.5% Triton X-100 in PBS. After labeling, sections were mounted in SlowFade Gold antifade reagent with DAPI (P36931, Thermo Fisher Scientific) for nuclei counterstaining. For co-labeling with rabbit anti-beta galactosidase (559761, MP Biomedicals; dilution 1:5,000 or former Cappel codice 559762 (Rabbit IgG)), free-floating brain sections were blocked and labeled with the respective primary and secondary antibodies in FBT blocking buffer (5% FBS, 1% BSA, 0.05% Tween 20, 10 mM Tris-HCl pH 7.5, 100 mM magnesium chloride in H_2_O). Imaging was performed on a STELLARIS 5 microscope (Leica; equipped with a 405-nm diode laser and a Leica white light laser (WLL; 485 nm to 685 nm excitation)) with a ×20 multi-immersion objective with 1,024 × 1,024 pixels. High-resolution images were acquired in frames with a line average of 16 or 32. Imaris imaging software v9.9 (Bitplane) was used for image processing and merging.

### Immunostaining of human brain tissue

Immunohistochemistry was performed on formalin-fixed paraffin-embedded tissue sections of human brain tissue (2–3 μm thick) as follows: for epitope demasking, deparaffinized sections were heated in a microwave histoprocessor (HistosPro) with DAKO Target Retrieval solution (citrate buffer pH 6.0 (S2367, DAKO) or pH 9.0 (S2367, DAKO)), washed with distilled water and transferred to 0.3% H_2_O_2_ for 10 min to block endogenous peroxidase. The sections were then washed with PBS and subsequently incubated with blocking buffer (5% NGS/PBS + 0.1% Triton) for at least 20 min. Thereafter, the sections were prepared for primary and secondary antibody incubation. The following primary antibodies were used (diluted in blocking solution): rabbit anti-IL-12Rβ2 (NBP1-85983, Novus Biologicals; 0.7 μg ml^−1^), rabbit IgG purified (PP64-10 KC, Merck; 0.7 μg ml^−1^) and anti-NeuN (MAB377, Chemikon, clone A60; dilution 1:100). Antibody binding was visualized using Dako EnVision + Dual Link System-HRP (DAB+) staining (DAKO) according to the manufacturer’s procedure. We recorded digital images of tissue sections using an Olympus BX41 light microscope with an Olympus ColorView IIIu camera and Olympus Cell B image acquisition software. For Immunofluorescence stainings, the endogenous enzyme blocking step with 0.3% H_2_O_2_ was omitted, and rabbit anti-IL-12Rβ2 (NBP1-85983, Novus Biologicals, dilution 1:1,000) was combined with mouse anti-Map2 (M4403, Sigma, clone HM-2, dilution 1:100) to co-label neurons. The secondary antibody mixture contained anti-rabbit Alexa Fluor 555 and anti-mouse Alexa Fluor 488 (all purchased from Thermo Fisher Scientific; dilution 1:250). Nuclear counterstaining was performed using SlowFade Gold antifade reagent with DAPI (Invitrogen). Imaging was performed on a STELLARIS 5 (Leica) microscope with a ×20 multi-immersion objective. Imaris imaging software v9.9 (Bitplane) was used for image processing.

### Quantitative histopathological analysis of spinal cords from mice with experimental autoimmune encephalomyelitis

Mice were euthanized through CO_2_ inhalation and transcardially perfused with ice-cold PBS. Spinal columns were carefully dissected out and fixed in 4% PFA (11762.00500, Morphisto) for 24 h at 4 °C, rinsed in PBS followed by decalcification in 0.5 M, pH 8, EDTA solution (A3145,0500, Axonlab) at 4 °C for five consecutive days while rotating. The spinal cords were then dissected into three parts (cervical, thoracic and lumbar) and cryoprotected in 30% sucrose in PBS at 4 °C for 24–72 h. Each spinal cord fraction was embedded coronally in Cryo Embedding Medium (41-3011-00, Medite) and frozen on dry ice. Frozen sections were cut at a 20–25-μm thickness using a Hyrax C60 cryostat (Zeiss). Subsequent blocking, staining steps and mounting were performed as described above. For the quantification of demyelination and inflammation, sequential spinal cord sections cut 200 μm apart were fluorescently labeled with rabbit anti-Iba1 (019-19741, Wako; dilution 1:200) and FluoroMyelin Green Fluorescent Myelin Stain (F34651, Thermo Fisher Scientific). For automated imaging, fluorochrome-labeled sections were acquired on a Zeiss Axio Scan. Z1 Slidescanner (Zen 2 software, blue edition) using a ×20 objective lens. The following fluorescence filters were used: AF405, AF488, AF546 or AF594 and AF647. The semiautomated quantification was performed on 20–30 images of *n* = 4 mice per group. Acquired images were cropped in Fiji to remove all pixels outside the spinal cord, and cropped images were then processed in Ilastik (v1.3.3)^61^.

Ilastik was trained to recognize lesions (DAPI bright, fluoromyelin low), WM (DAPI low, fluoromyelin high), GM (DAPI low, fluoromyelin low) and background (no signal), and to calculate the probability that each pixel belonged to each category. These probabilities were exported to Fiji as 8-bit images using the default algorithm. The areas of lesions and WM were then measured, and the percentage of demyelination was calculated as follows: 100 × area of lesions / (area of lesions + area of WM). We report the average percentage demyelination of each mouse over the total number of sections.

### Bulk RNA isolation of primary neuronal cultures

Dissociated cerebellar cells were prepared and maintained as described above. At DIV 14, primary neuronal cells were stimulated with 30 ng ml^−1^ or 100 ng ml^−1^ of Recombinant Murine IL-12p70 (210-12, PeproTech) for 18 h. Cerebellar neurons were harvested and lysed in 300 μl RLT buffer of the RNeasy Micro Kit (74004, Qiagen) and total RNA was extracted according to the manufacturers’ protocol.

### Library preparation, cluster generation and next-generation sequencing

The quality of the isolated RNA was determined with a Fragment Analyzer (Agilent). The TruSeq Stranded mRNA (Illumina) was used in the succeeding steps. Briefly, total RNA samples (100–1,000 ng) were subjected to poly(A) enrichment and then they were reverse transcribed into double-stranded cDNA. The cDNA samples were fragmented, end-repaired and adenylated before ligation of TruSeq adaptors containing unique dual indices for multiplexing. Fragments containing TruSeq adaptors on both ends were selectively enriched with PCR. The quality and quantity of the enriched libraries were validated using the Fragment Analyzer (Agilent). The product is a smear with an average fragment size of approximately 260 bp. The libraries were normalized to 10 nM in Tris-Cl 10 mM, pH8.5 with 0.1% Tween 20. The NovaSeq 6000 (Illumina) was used for cluster generation and sequencing according to standard protocol. Sequencing configuration produced single-end reads of 100 bp.

### Bulk transcriptome analysis of primary neuronal cultures

The raw reads were aligned to mouse genome build GRCm39 using the STAR aligner v2.7.8a, with FeatureCounts used to calculate read counts per gene based on GENCODE gene annotation release M26. Counts were counts-per-million normalized, log_2_ transformed and only genes were retained if expressed at one count per million in at least two samples. PCA was computed using the BiocGenerics package after variance stabilizing transformation using the DEseq2 package^62^ (1.38.3) and visualized using ggplot2. DEGs between conditions were computed using gene-wise negative binomial generalized linear models as implemented in the edgeR package^63^ (v3.40.2) and applying a Benjamini–Hochberg correction to account for multiple testing. Heat maps of DEGs were generated using the pheatmap package.

Differential analysis of modular gene coexpression was performed using the R implementation of CEMiTool using default parameters^64^. For gene-set enrichment analysis and interactome integration, murine gene names were converted to human orthologs using biomaRt. Gene-set enrichment analysis of gene modules was performed using the hallmark gene-set collection from MSigDB^65^.

Interactome data from the STRING database version 11 was integrated after filtering on interactions with a minimum score of 200 (ref. ^66^) to obtain gene coexpression and interaction networks for each module.

### Single-nuclei preparation and fluorescence-activated nuclei sorting

Single-nuclei suspensions were prepared as described before^12^. Mouse cerebellum and brainstem (of one brain hemisphere) and cervical spinal cord (C1-C2) were collected from adult male mice (8–12 weeks old) and immediately snap-frozen in liquid nitrogen and stored at −80 °C until further processing. Nuclei were isolated with the EZ PREP lysis buffer (NUC-101, Sigma). Tissue samples were homogenized using a glass dounce tissue grinder (D8938, Sigma; 25 strokes with pastel A, 25 strokes with pastel B) in 2 ml of ice-cold EZ prep lysis buffer and incubated on ice with an additional 2 ml of ice-cold EZ PREP lysis buffer. During incubation, 1 μΜ of Hoechst (H3570, Thermo Fisher Scientific) dye and 40 U μl^−1^ of RiboLock inhibitors (EO0382, Thermo Fisher Scientific) were added to the homogenate. Following incubation, the homogenate was filtered through a 30-μm FACS tube filter. Nuclei were sorted based on the fluorescence Hoechst signal using a BD FACS Aria sorter III with an 85-μm nozzle configuration at 4 °C, directly into PBS + 4% BSA + RiboLock inhibitors (40 U μl^−1^; EO0382). As CNS nuclei vary strongly in size, no doublet exclusion was performed based on FSC or SSC to avoid bias against nucleus size. Nuclei were then counted based on brightfield image and Trypan Blue staining (15250061) using a Neubauer counting chamber and a Keyence BZX-710 microscope.

### Droplet-based single-nucleus RNA sequencing

RNA-seq of single nuclei was performed in two independent batches (batch 1, *n* = 1 *Nestin*^Cre/+^*Il12rb2*^fl/fl^ and *n* = 1 *Il12rb2*^fl/fl^; batch 2, *n* = 3 *Nestin*^Cre/+^*Il12rb2*^fl/fl^ and *n* = 3 *Il12rb2*^fl/fl^). Immediately after extraction, 17,500 sorted nuclei per sample were loaded onto a Chromium Single Cell 3′ Chip (10x Genomics) and processed for the single-nucleus cDNA library preparation (Chromium Next GEM Single Cell 3′ Reagent Kits v3.1 protocol). Around 50,000 reads per nucleus were sequenced using the Illumina NovaSeq 6000 no. 1 platform according to the manufacturer’s instructions without modifications (R1 = 28, i7 = 10, i5 = 10, R2 = 90). Preparation of cDNA libraries and sequencing were performed at the Functional Genomics Center Zurich. Cell Ranger software (v6.0.2) was implemented for library demultiplexing, barcode processing, fastq file generation, gene alignment to the mouse genome (GENCODE reference build GRCm39) and UMI counts. We implemented the include-introns option for counting intronic reads, as the snRNA-seq assay captures unspliced pre-mRNA as well as mature mRNA. For each sample, a Cell Ranger report was obtained with all the available information regarding sequencing and mapping parameters. All samples were merged into a matrix using Cell Ranger (cellranger --aggr function).

### Quality control and data pre-processing

Starting from the filtered gene-cell count matrix produced by Cell Ranger’s built-in cell calling algorithms, we proceeded with the SCANPY (v1.8.2) workflow in Python^67^. Doublet exclusion was used individually for each sample using the Scrublet^68^ package with variable thresholds. Quality-control processing was performed individually for each batch. Low-quality nuclei were filtered out based on the number of unique genes (<500 and <200 in batch 1 and batch 2, respectively), total UMIs (<6,000) and fraction of mitochondrial counts (<5%). Genes present in less than three nuclei were removed. Counts were log1p transformed normalized to 10,000 counts and scaled for visualization purposes. Individual batches were integrated into a combined SCVI^69^ model consisting of two layers and 30 latent dimensions using the 3,000 most variable features.

### Dimensionality reduction, clustering visualization and cell-type identification

Dimensionality reduction using UMAP was used within the SCANPY framework. For this, the neighbor graph was constructed from the combined SCVI latent space. Community detection using Leiden was applied for cell-type classification. For assigning clusters to cell types, we defined a list of marker genes per cluster and ranked genes applying the Wilcoxon rank-sum test. The assignment of cell-type identity to clusters was based on known linage markers in line with previously published snRNA-seq^16^ studies and atlases^70,71^.

### Differential gene expression and downstream analysis

For differential expression between *Il12rb2*^fl/fl^ and *Nestin*^Cre/+^*Il12rb2*^fl/fl^ mice, all genes per cell type were tested. DEGs were identified using a non-parametric Wilcoxon rank-sum test and Benjamini–Hochberg correction was applied to correct for multiple testing. For pseudo-bulk analysis, the sum of the transcripts for all cells within a given cluster of a sample was computed. Sample-level differential expression was carried out using the DEseq2 package^62^. Gene signatures were manually curated (Supplementary Table 2) and were computed as described before^72^. Bar graphs, volcano plots and lollipop plots were drawn in ggplot2. Heat maps were drawn using the pheatmap package. Dot plots were drawn using the Seurat framework^73^, in SCANPY^67^ or using ggplot2. Violin plots and scatterplots were drawn in SCANPY.

### Analysis of ligand–receptor interactions

Ligand–receptor interaction analysis was carried out using NicheNet^24^. Potential ligand–receptor pairs were restricted to pairs in which the ligand is expressed in at least 10% of the sender cells and the receptor is present in at least 10% of receivers. Sender cells were defined based on their ability to express *Il12rb2* and demonstrate transcriptional changes after genetic deletion using *Nestin*^Cre/+^*Il12rb2*^fl/fl^ mice (MOL1, MOL2, excitatory neurons, cholinergic neurons, granule cells and MLIs). For each receiver cell, NicheNet predicted the ligands that best explained DEGs (adjusted *P* value < 0.05 and abs(log2FC) > 0.5) upon *Il12rb2* deletion. Top-ranking predicted ligands and the corresponding target genes for each receiver cell type were visualized in circos plots using the circlize package. Dot plots showing the respective ligand expression across sender cells were generated in ggplot2. Heat maps of top-ranking predicted ligands in sender cells and corresponding receptors in recipients were generated using the pheatmap package.

### Reanalysis of single-nucleus RNA sequencing of multiple sclerosis tissue

Publicly available gene expression matrix data from Absinta et al. (accessed at GSE180759; five individuals with progressive MS and three age-matched and sex-matched non-affected, non-dementia controls)^13^ were reanalyzed for the expression of *IL12RB1* and *IL12RB2*. We downloaded the available gene expression matrix, and downstream analysis was performed by implementing the Seurat v4 R package^73^.

In brief, the filtered raw count matrix was log-normalized within each nucleus and included *n* = 66,432 nuclei. The top 2,000 variable genes calculated by Seurat were used to harmonize and integrate sample datasets by individual (FindIntegrationAnchors and IntegrateData functions). On scaled data, linear dimension reduction (PCA) was performed, and the top 30 principal components were implemented for the unsupervised clustering. The assignment of cell type was based on the original clustering annotation provided.

We further downloaded raw snRNA-seq data available (Sequence Read Archive accession number PRJNA544731) from ref. ^14^, in which nuclei were extracted from entire MS tissue sections (*n* = 12 cases) and compared to similarly processed control brain tissue (*n* = 9 cases). We processed the raw data, starting from the library demultiplexing, as described above for our own data. Cell Ranger software was implemented for library demultiplexing, barcode processing, fastq file generation, gene alignment to the human genome (GRCh38-3.0 human genome with the intronic reads option) and UMI counts. Each sample was individually filtered based on Seurat guidelines for snRNA-seq, and only nuclei with <5% of mitochondrial contamination and between 200 and 2,500 genes expressed were retained for further analysis, reducing the probability of doublets.

The Seurat function SCTransform (v2)^74^ was used to normalize the datasets, FindIntegrationAnchors was used to identify anchors between datasets and IntegrateData was used to run an integrated analysis. DEGs were defined as *P* < 0.05 (*P* adjusted for multiple comparisons) in ≥25% of cell populations and showing, on average, a >0.25-fold difference (log scale) between groups of cells.

### Fluorescence-activated nuclei sorting for bulk RNA isolation

Nuclei of murine brains and spinal cords were isolated and sorted as described above. For specific labeling of neuronal and oligodendrocyte nuclei, the homogenates were directly stained with Hoechst, NeuN (ab190195, clone EPR12763, Abcam; dilution 1:200) and Olig2 (ab225100, clone EPR2673, Abcam; dilution 1:200). Hoechst^+^NeuN^+^ and Hoechst^+^Olig2^+^ populations were directly sorted into RLT Lysis buffer (74004, Qiagen) and further processed according to the manufacturer’s instructions.

### High dimensional analysis of flow cytometry data

Raw flow cytometry data were pre-processed in FlowJo (Tree Star). Compensated and cleaned data were exported to Rstudio version 4.0.1. After transformation and percentile normalization, UMAPs^75^ were generated using the package ‘umap’ version 0.2.7.0, and FlowSOM^76^ version 2.6.0 metaclusters were merged and annotated based on the median expression profile and their localization on the UMAP. Heat maps were generated using the pheatmap’ package version 1.0.12. All remaining plots were generated using the ‘ggplot2*’* package version 3.3.5.

### Statistical analysis

Statistical analysis was carried out using Prism 9 (GraphPad Software). Statistical significance of in vivo experiments (clinical scores over time) was determined with a repeated-measures two-way analysis of variance (ANOVA) or mixed-effects model (restricted maximum likelihood) when values were missing, followed by Bonferroni or Sidak multiple-comparison test. To test if the data were normally distributed, the Shapiro–Wilk test or Kolmogorov–Smirnov test were used and the quantile–quantile plots were examined. Group means were compared with two-tailed, unpaired *t*-tests, Mann–Whitney tests and one-way ANOVA or Kruskal–Wallis tests, corrected for multiple comparisons using the Dunn’s or Bonferroni test.

Statistical analysis of CNS infiltration of different cell types between different groups was carried out in Rstudio (version 4.0.1) using *t*-tests. *P* values below 0.05 were considered as significant and are indicated by asterisks (*P < 0.05, **P < 0.01, ***P < 0.001; ****P < 0.0001) or numerical values on the respective graphs and figure legends. *N* represents the number of biologically independent animals. In all cases, data are shown as the mean ± s.e.m., unless otherwise indicated. Cohen’s *d* effect size estimates were calculated using the ‘effsize’ package. The Wilcoxon rank-sum test was applied to calculate statistical differences among human snRNA-seq cell populations.

Unless otherwise stated, we compare genetically modified and littermate control mice that were weaned and randomly allocated to their housing cages by animal caretakers who were not involved in the experimental planning. The distribution of the genotype was determined by the Mendelian ratio (assumed to be the case for all strains), and selection for the experimental cohort was not influenced by the researcher, thereby randomized. Simultaneously, mice of different genotypes were randomly co-housed—controlling for environmental bias, for example, genotype-specific microbiome. Data collection was randomized using a computer randomization process (R). Scoring, data collection and analysis of all experiments was performed in a blinded manner. Blocking was used to control for sex, age and litter in the experimental design.

No animals or data points were excluded. Animals that met withdrawal criteria were euthanized and scores were only recorded from the surviving animals. To minimize attrition bias, we reported the cumulative clinical score per day.

**Table 1:** Capability of IL-12 sensing in bone marrow chimera mice

	*Il12rb2*^del^ → *Il12rb2*^del^	*Il12rb2*^fl^ → *Il12rb2*^del^	*Il12rb2*^del^ → *Il12rb2*^fl^	*Il12rb2*^fl^ → *Il12rb2*^fl^
Peripheral and CNS-invading leukocytes	−	+	−	+
CNS tissue-resident cells (neuroectoderm, microglia and T_RM_ cells)	−	−	+	+
EAE outcome	Hyperinflamed	Hyperinflamed	Normal	Normal

+, IL-12 responsive, −, IL-12 unresponsive.

### Reporting summary

Further information on research design is available in the Nature Portfolio Reporting Summary linked to this article.

## Online content

Any methods, additional references, Nature Portfolio reporting summaries, source data, extended data, supplementary information, acknowledgements, peer review information; details of author contributions and competing interests; and statements of data and code availability are available at 10.1038/s41593-023-01435-z.

### Supplementary information

Reporting Summary

Supplementary Tables 1–4.

### Source data

Source Data Fig. 1 Statistical source data.

Source Data Fig. 2 Statistical source data.

Source Data Fig. 3 Statistical source data.

Source Data Fig. 5 Statistical source data.

Source Data Extended Data Fig. 1 Statistical source data.

Source Data Extended Data Fig. 2 Statistical source data.

Source Data Extended Data Fig. 3 Statistical source data.

Source Data Extended Data Fig. 7 Statistical source data.

Unmodified Gels Extended Data Fig. 2 Full-length, unprocessed gels or blots.

## Data Availability

The data that support the findings of this study are available from the corresponding authors upon request. All raw sequencing data generated in this study have been deposited at NCBI’s Gene Expression Omnibus repository and are accessible under accession numbers GSE236464 (next-generation sequencing) and GSE236540 (snRNA-seq). The raw count file and steps to reproduce the next-generation sequencing analysis are available at https://data.mendeley.com/datasets/rh4zjz8vt3/1/. Raw snRNA-seq counts, including relevant metadata and steps to reproduce the snRNA-seq analysis are available at 10.17632/zgr9bj57r4.2. Data from ref. ^13^ were accessed at GSE180759. Data from ref. ^14^ were accessed at the Sequence Read Archive under accession number PRJNA544731. This study did not generate new or unique reagents. Source data are provided with this paper.

## Code availability

No custom software was developed during this study. Open-source packages and libraries and corresponding versions that have been utilized during the computational analysis are described in Methods and Reporting Summary. Notebooks used for the analysis are available upon request.
