# Supplementary material for: IL-12 sensing in neurons induces neuroprotective CNS tissue adaptation and attenuates neuroinflammation in mice
Source: Nat Neurosci. 2023 Sep 25;26(10):1701–12. doi: 10.1038/s41593-023-01435-z (PMC10545539; doi:10.1038/s41593-023-01435-z)
Supplement: Supplementary file 1 — Reporting Summary [file 41593_2023_1435_MOESM1_ESM.pdf]

nature portfolio

Corresponding author(s): Burkhard Becher, Sarah Mundt

Last updated by author(s): Aug 9, 2023

Reporting Summary

Nature Portfolio wishes to improve the reproducibility of the work that we publish. This form provides structure for consistency and transparency in reporting. For further information on Nature Portfolio policies, see our Editorial Policies and the Editorial Policy Checklist.

Statistics

For all statistical analyses, confirm that the following items are present in the figure legend, table legend, main text, or Methods section.

n/a	Confirmed	
☐	☒	The exact sample size (*n*) for each experimental group/condition, given as a discrete number and unit of measurement
☐	☒	A statement on whether measurements were taken from distinct samples or whether the same sample was measured repeatedly
☐	☒	The statistical test(s) used AND whether they are one- or two-sided<br>*Only common tests should be described solely by name; describe more complex techniques in the Methods section.*
☒	☐	A description of all covariates tested
☐	☒	A description of any assumptions or corrections, such as tests of normality and adjustment for multiple comparisons
☐	☒	A full description of the statistical parameters including central tendency (e.g. means) or other basic estimates (e.g. regression coefficient) AND variation (e.g. standard deviation) or associated estimates of uncertainty (e.g. confidence intervals)
☐	☒	For null hypothesis testing, the test statistic (e.g. *F*, *t*, *r*) with confidence intervals, effect sizes, degrees of freedom and *P* value noted<br>*Give P values as exact values whenever suitable.*
☒	☐	For Bayesian analysis, information on the choice of priors and Markov chain Monte Carlo settings
☒	☐	For hierarchical and complex designs, identification of the appropriate level for tests and full reporting of outcomes
☐	☒	Estimates of effect sizes (e.g. Cohen's *d*, Pearson's *r*), indicating how they were calculated

Our web collection on statistics for biologists contains articles on many of the points above.

Software and code

Policy information about availability of computer code

Data collection

• Spectral flow cytometry: 5L Cytek® Aurora; SpectroFlo® v2 Software
• Flow cytometry:
FACS Symphony A5, BD Biosciences; FACS Diva Software v9.1
LSRII Fortessa, BD Biosciences; FACS Diva Software v9
• Fluorescence Activated Cell Sorting: BD FACSAria™ III; FACSDIVA Software v9
• Quantitative RT-PCR: CFX384 Touch Real-Time PCR Detection System (Bio-Rad)
• Western Blot: Odyssey® CLx Imager
• Confocal Laser Scanning Microscopy:
- Leica STELLARIS 5 confocal microscope, equipped with 405 nm diode laser and a Leica white light laser (WLL- 485 nm to 685 nm excitation)
- Leica TCS SP5 confocal laser scanning microscope; LAS AF scan software v.4.0 (Leica Microsystems)
• Widefield Microscopy:
- Zeiss Axio Scan. Z1 Slidescanner; Zen 2 software, blue edition
• Immunohistochemistry: Olympus BX41 light microscope with an Olympus ColorView IIIu camera
• Droplet based Single Nucleus RNA sequencing: Murine CNS nuclei were loaded onto a Chromium Single Cell 3′ Chip (10X Genomics) and processed for the single-nucleus cDNA library preparation (Chromium Next GEM Single Cell 3′ Reagent Kits v3.1 protocol). 50.000 reads per nucleus were sequenced using the Illumina Novaseq 6000 #1 platform according to the manufacturer's instructions without modifications (R1= 28, i7= 10, i5= 10, R2=90).
• Next Generation Sequencing: Novaseq 6000 (Illumina, Inc, California, USA)
• Protein concentration and ELISA analysis: Photometer Tecan Infinite®200M

Data analysis

• R version 4.0.1 was used in Rstudio 4.0.1 for downstream data analyses. FlowJo software (version 10.8.0, Tree Star Inc.) was used for

Data analysis

manual gating and data pre-processing. CellRanger v6.0.2 (snRNAseq) and STAR aligner v2.7.8a (bulk RNAseq) were used to process raw sequencing reads. For downstream analysis the following packages were used as indicated in the respective methods section of the manuscript: umap (v0.2.7.0), FlowSOM (v 2.6.0), ggplot2 (v.3.3.5), pheatmap (v1.0.12), Seurat (v4.0), SCANPY (v1.8.2), stats (v4.0.1), edgeR (v3.40.2), DEseq2 (v1.38.3).
• GraphPad Prism v9 (GraphPad Software, Inc.) was used for visualization and statistical analysis of clinical EAE data, RT-qPCR data and manually gated flow cytometry data (only for bone-marrow chimera flow cytometry data).
• Imaris imaging software v9.9 (Bitplane, Zurich, Switzerland) was used for image processing and merging. Quantitative histopathological analysis was performed using Ilastik (v1.3.3) and Fiji. Western blot images were processed and analyzed using ImageJ (v1.53t).

No custom software has been developed during this study. Open source packages and libraries and corresponding versions that have been utilized during the computational analysis are described in the method section of the manuscript and the reporting summary. Notebooks used for the analysis are available upon request.

For manuscripts utilizing custom algorithms or software that are central to the research but not yet described in published literature, software must be made available to editors and reviewers. We strongly encourage code deposition in a community repository (e.g. GitHub). See the Nature Portfolio guidelines for submitting code & software for further information.

Data

Policy information about availability of data

All manuscripts must include a data availability statement. This statement should provide the following information, where applicable:

- Accession codes, unique identifiers, or web links for publicly available datasets
- A description of any restrictions on data availability
- For clinical datasets or third party data, please ensure that the statement adheres to our policy

All raw sequencing data generated in this study have been deposited at NCBI's Gene Expression Omnibus (GEO) repository and are accessible through GEO Series accession number GSE236464 (Next Generation Sequencing) and GSE236540 (snRNA-seq).

The raw count file and steps to reproduce the NGS analysis are available at https://data.mendeley.com/datasets/rh4zjz8vt3/1.
Raw snRNA-seq counts, including relevant metadata and steps to reproduce the snRNA-seq analysis are available at https://doi.org/10.17632/zgr9bj57r4.2.

Data from Absinta et al., 2021 were accessed at GSE180759.
Data from Schirmer et al., 2019 were accessed at SRA accession number PRJNA544731.

All other data are available in the main text or the supplementary materials.

Human research participants

Policy information about studies involving human research participants and Sex and Gender in Research.

Reporting on sex and gender

Sex and gender information has not been collected and does not influence the results.

Population characteristics

Post-mortem sections from three patients with Multiple Sclerosis were obtained from the archives of the Institute of Neuropathology at the University Hospital Zurich, Switzerland

The MS patients (n=3) whose brain tissue samples were examined were all females, and between 64 - 68 years of age. The year of MS diagnosis was in the 1970`s (n=2) and in the early 1990`s (n=1), respectively. One secondary-progressive MS patient was previously treated with interferons and mitoxantrone, but not at the time of death. For the two other patients (with chronic MS lesions/ chronic-progressive MS course) there was no specific MS treatment documented in the autopsy report. Autopsies were performed between 2016 – 2017, and causes of death were documented as: acute hypoxic brain injury, aortic dissection and pneumonia.

Recruitment

No donors were recruited. Informed consent for autopsy was given by the next-of-kin in all cases.

Ethics oversight

Case series do not need institutional review board approval according to Swiss legislation.

Note that full information on the approval of the study protocol must also be provided in the manuscript.

Field-specific reporting

Please select the one below that is the best fit for your research. If you are not sure, read the appropriate sections before making your selection.

☒ Life sciences ☐ Behavioural & social sciences ☐ Ecological, evolutionary & environmental sciences

For a reference copy of the document with all sections, see nature.com/documents/nr-reporting-summary-flat.pdf

Life sciences study design

All studies must disclose on these points even when the disclosure is negative.

Sample size	For the sample size calculation we used an alpha = 0.05 and a power of 0.8. We used data from previous experiments for the mean and Standard deviation to calculate the standardized effect size using G Power. We determined the required sample size to be able to see a 30% difference in the cumulative score (AUC) for disease course and 70% by qPCR/ gene expression for assessing the targeting efficacy/specificity of our strains.<br><br>snRNA-seq: More than 10,000 nuclei (n=4) per sample were sequenced (10X Genomics). Details are provided in the respective text, figure legends and methods section.
Data exclusions	No animals were excluded from downstream analysis.<br>For spectral flow cytometry, we pregated on single, live CD45+ cells for downstream analysis in R studio.<br>For single nucleus RNA sequencing data analysis, nuclei exclusion was done on the basis of QC and is described in detail in the methods.<br>Initially, snRNA seq data of a NestinCre_Il12rb2fl mouse and Il12rb2fl control has been generated during steady state. However, as initial analyses showed no marked differences between nuclei derived from both genotypes, we excluded steady state data from the manuscript and repeated the sequencing for EAE only.
Replication	All experiments (except targeting experiments) were performed at least twice. The precise number of experiments is indicated in the figure legends.<br><br>Bulk RNAseq of primary murine neuronal cultures was performed in n=5 replicates per group.<br><br>snRNA-seq: We performed three independent sequencing experiments and successfully sequenced 11 samples.<br>The first experiment included two samples: Il12rb2fl_naive and NestinCre_Il12rb2fl_naive. The second experiment included three samples: Il12rb2fl_EAE, NestinCre_Il12rb2fl_EAE and Il12rb2_naive_AUG. Sample Il12rb2_naive_AUG was only an internal control to assess reproducibility and batch effects between the two sequencing experiments and is excluded from the final visualizations displayed in our manuscript.<br>Since initial analyses indicated that genotype differences were predominantly present in early onset EAE, we performed the validation only for EAE samples and excluded the initial steady state data from the manuscript.<br><br><br>The third experiment included six samples: Il12rb2fl/fl EAE (n=3) and NestinCreIl12rb2fl/fl (n=3) EAE.<br>Across the manuscript we only refer to the two batches of EAE samples (steady state samples were not included in the analysis).
Randomization	Unless otherwise stated, we compare genetically modified and littermate control mice which have been weaned and randomly allocated to their housing cages by animal caretakers who are not involved in the experimental planning. The distribution of the genotype is determined by the Mendelian ratio (assumed to be the case for all strains) and selection for the experimental cohort was not influenced by the researcher, thereby randomized. Simultaneously, mice of different genotypes are randomly cohoused - controlling for environmental bias e.g. genotype specific microbiome. Data collection was randomized using a computer randomization process (R).
Blinding	Data collection and analysis of all experiments was performed in a blinded manner.

Reporting for specific materials, systems and methods

We require information from authors about some types of materials, experimental systems and methods used in many studies. Here, indicate whether each material, system or method listed is relevant to your study. If you are not sure if a list item applies to your research, read the appropriate section before selecting a response.

Materials & experimental systems

n/a	Involved in the study	
☐	☒	Antibodies
☒	☐	Eukaryotic cell lines
☒	☐	Palaeontology and archaeology
☐	☒	Animals and other organisms
☒	☐	Clinical data
☒	☐	Dual use research of concern

Methods

n/a	Involved in the study	
☒	☐	ChIP-seq
☐	☒	Flow cytometry
☒	☐	MRI-based neuroimaging

Antibodies

Antibodies used	Spectral Flow Cytometry: CD8 (#564920, BD Biosciences, clone 53-6.7, BUV805, dilution 1:100), CD274 (#124311, Biolegend, clone 10F.9G2, APC, dilution 1:200), CD274 (#25-5982-82, eBioscience, clone MIH5, PE-Cy7, dilution 1:500), CD45R (#552094, BD Biosciences, clone RA3-6B2, APC-Cy7, dilution 1:200), CD38 (#102714, Biolegend, clone 90, AF488, dilution 1:300), Granzyme B (#515406, Biolegend, clone GB11, AF647, dilution 1:100), I-A / I-E (#107622, Biolegend, clone M5/114.15.2, AF700, dilution 1:400), Siglec-F (#564514, BD Biosciences, clone E50-2440, BB515, dilution 1:400), NK1.1 (#566502, BD Biosciences, clone PK136, BB700,

dilution 1:100), CD45 (#564279 , BD Biosciences, clone 30-F11, BUV395, dilution 1:200), CD4 (#564667, BD Biosciences, clone GK1.5, BUV496, dilution 1:200), Ly6G (#565707, BD Biosciences, clone 1A8, BUV563, dilution 1:100), CD19 (#565076, BD Biosciences, clone 1D3, BUV661, dilution 1:200), CD44 (#612799 , BD Biosciences, clone IM7, BUV737, dilution 1:200), CD64 (#139309, Biolegend, clone X54-5/7.1, BV421, dilution 1:100), Ki67 (#566109, BD Biosciences, clone B56, BV480, dilution 1:100), Ki67 (#652420, Biolegend, clone 16A8, AF700, dilution 1:200), F4/80 (#MCA497A488, AbD Serotec, clone BM8, BV510, dilution 1:50), F4/80 (#MCA497A488, AbD Serotecclone, clone CI:A3-1, AF647, dilution 1:200), CD62L (#104433, Biolegend, clone MEL-14, BV570, dilution 1:100), CX3CR1 (#149027, Biolegend, clone SA011F11, BV605, dilution 1:400), CD11b (#101239, Biolegend, clone M1/70, BV650 dilution 1:400), Ly-6C (#128037, Biolegend, clone HK1.4, BV711, dilution 1:400), MerTK (#78-5751-82, eBioscience, clone DS5MMER, SuperBright780, dilution 1:50), CD103 (#121406, Biolegend, clone 2E7, PE, 1:100), TCR beta chain (#109209, Biolegend, clone H57-597, PE-Cy5, dilution 1:400), CD11c (#35-0114-82, eBioscience, clone N418, PE-Cy5.5, 1:400), CD49d (#103618, Biolegend, clone R1-2, PE-Cy7, dilution 1:100), Foxp3 (#61-5773-82, eBioscience, clone FJK-16s, PE-eFlour610, dilution 1:200), CD90.2 (#105324, Biolegend, clone 30-H12, Pacific Blue, dilution 1:200), TCRγδ (#46-5711-82, eBioscience, clone GL3, PerCP-eFlour710, dilution 1:100), CD88 (#135811, Biolegend, clone 20/70, Biotin, dilution 1:200), Arginase-1 (#17-3697-82, Invitrogen, clone A1exF5, APC, dilution 1:200), IL-1 beta Pro-form (#12-7114-82, eBioscience, clone NJTEN3, PE, dilution 1:200), CD14 (#64-0141-82, Invitrogen, clone Sa2-B, SuperBright 645, dilution 1:200), CD49b (#108918, Biolegend, clone DX5, Pacific Blue, dilution 1:200), Streptavidin (#564923, BD Biosciences, BUV805- conjugated, dilution 1:200), NKp46 (#46-3351-82, eBioscience, clone 29A1.4 PerCP-eFlour710, dilution 1:200), NK1.1 (#108749, Biolegend, clone PK136, BV785, dilution 1:200), Ly-6C (#553104, BD Biosciences, clone AL-21, FITC, dilution 1:400), CD11c (#117308, Biolegend, clone N418, PE, dilution 1:200), CD45R (#562290, BD Biosciences, clone RA3-6B2, PE-CF594, dilution 1:400), F4/80 (123112#,Biolegend, clone BM8, PE-Cy5, dilution 1:400).
Dead cell exclusion:Fixable Viability Kit (Near-IR staining, dilution 1:1000, Biolegend)

Cell-sorting: CD45 (#103126, Biolegend, clone 30-F11, Pacific Blue, dilution 1:600), CD11b (#101263, Biolegend, clone M1/70, BV510, dilution 1:400), CD44 (#103049, Biolegend, clone IM7, BV650, dilution 1:400), CX3CR1 (#149027, Biolegend, clone SA011F11, BV605, dilution 1:400), LY6C (#128037, Biolegend, clone HK1.4, BV711, dilution 1:400), LY6G (#127606, Biolegend, clone 1A8, FITC, dilution 1:400), NK1.1 (#25-5941-82, eBioscience, clone PK136, PE-Cy7, dilution 1:400), O4 (#130-117-711, Miltenyi, clone REA576, PE, dilution 1:25), ACSA-2 (#130-116-245, Miltenyi, REA969, APC, dilution 1:25), CD88 (#135811, Biolegend, clone 20/70, Biotin, dilution 1:200), F4/80 (#MCA497A488, AbD Serotec, clone CI:A3-1, AF647, dilution 1:200), CD140a (#135911, Biolegend, clone APA5, PE-Cy7, dilution 1:400), TER119 (#17-5921-81, Biolegend, clone Ter-119, APC, dilution 1:200), CD3 (#100236, Biolegend, clone 17A2, APC, dilution 1:200), CD31 (#102507, Biolegend, clone MEC13.3, PE, dilution 1:200), CD31 (#102427, Biolegend, clone 390, BV605, dilution 1:400), CD4 (#100548, Biolegend, RM4-5, BV605, dilution 1:400), Streptavidin (#557598, BD Biosciences, PE-Cy7- conjugated, dilution 1:400), GalC (#FCMAB312F, Milli-Mark, FITC, clone mGalC, dilution 1:10).

Dead cell exclusion: Fixable Viability Kit (Near-IR staining, dilution 1:500, Biolegend).

Mouse immunofluorescence: rat anti-GFAP (#13-0300, Thermo Fisher Scientific; 1:400), rabbit anti-Calbindin (##ab108404, clone EP3478, Abcam; 1:500), mouse anti-NeuN (#MAB377, clone A60, Chemikon 1:500), mouse CC1 Anti-APC (Ab-7) (#OP80-100UG, Merck), mouse anti-Olig2 (#66513-1-IG, Thermo Fisher Scientific; 1:200), rabbit-anti-beta galactosidase (#559761, MP Biomedicals; 1:5000 or former Cappel codice 559762 (Rabbit IgG)), rabbit anti-Iba1 (#019-19741, Wako; 1:200) and FluoroMyelin™ Green Fluorescent Myelin Stain (#F34651, 1:200, Thermo Fisher Scientific).
Fluorochrome - conjugated secondary antibodies: goat anti-mouse/anti-rabbit/anti-rat, streptavidin etc, conjugated to AlexaFluor 488, 594, 633, 647, all purchased from ThermoFisher Scientific; 1:500

Human immunohistochemistry: rabbit anti-IL-12Rβ2 (#NBP1-85983, Novus Biologicals; 1:1000), rabbit IgG purified (#PP64-10 KC, Merck; 0.7µg/ml) and anti-NeuN (#MAB377, clone A60, Chemikon 1:100).

Human immunofluorescence: rabbit anti-IL-12Rβ2 (#NBP1-85983, Novus Biologicals, 1:1000), mouse anti-Map2 (#M4403, Sigma, clone HM-2, 1:100)
Fluorochrome - conjugated secondary antibodies: anti-rabbit AlexaFluor-555 and anti-mouse AlexaFluor-488 (all purchased from ThermoFisher Scientific; 1:250)

Western Blot: Cell Signaling: rabbit anti-mouse Stat4 #2653, 1:2000; rabbit anti-mouse Phospho-Stat4-Tyr693 #4134, 1:2000; mouse monoclonal anti-actin, #MAB1501, Merck, 1:5000;
Goat Anti-Rabbit IgG H&L (HRP) (#ab6721, Abcam; 1:20,000);
Alexa Fluor® 680 AffiniPure Donkey Anti-Mouse IgG (H+L) (#715-625-151, Jackson ImmunoResearch; 1:10,000)

Fluorescence Activated Nuclei Sorting: NeuN (#ab190195, Abcam;1:200); Olig2 (ab225100, Abcam;1:200); Hoechst dye (#H3570, Thermo Fisher Scientific)

NK cell depletion: anti-NK1.1 mAb (# BP0036, clone PK136, BioXcell)

Validation

All antibodies used in our study are commercially available and validated by the manufacturer (https://www.biolegend.com/en-us/quality/quality-control;
https://www.thermofisher.com/ch/en/home/life-science/antibodies/invitrogen-antibody-validation.html;
https://www.bdbiosciences.com/en-eu/products/reagents/flow-cytometry-reagents;
https://www.miltenyibiotec.com/CH-en/products/macs-antibodies/Antibody-production-development-and-quality-control.html).

All primary antibodies for mouse and human immunofluorescence are commercially available and validated by the manufacturer (https://www.thermofisher.com/ch/en/home/life-science/antibodies/invitrogen-antibody-validation.html;
https://www.abcam.com/primary-antibodies/how-we-validate-our-antibodies;
https://www.merckmillipore.com/CH/de/life-science-research/antibodies-assays/antibodies-overview/Antibody-Development-and-Validation/cFOb.qB.8McAAAFOb64qQvSS,nav;
https://www.novusbio.com/5-pillars-validation;
https://labchem-wako.fujifilm.com/us/product/detail/W01W0101-1974.html;
https://www.mpbio.com/ca/rabbit-igg-fraction-to-beta-galactosidase#documents and https://www.mpbio.com/ca/coa/)
For mouse and human immunofluorescence, staining with secondary antibody alone was used as a negative control in order to rule

out non-specific staining. Labeling was compared to the staining patterns reported by the manufacturer.

Animals and other research organisms

Policy information about studies involving animals; ARRIVE guidelines recommended for reporting animal research, and Sex and Gender in Research

Laboratory animals	Species: Mus musculus. Strains: Knockout-first (promoter-driven) Il12rb2fl/fl and Il12rb2LacZ mice were generated at the Institute of Laboratory Animal Science (LTK) of the University of Zurich. B6.C-Tg(CMV-Cre)1Cgn/J (The Jackson Laboratory, #006054); B6.Cg-Tg(Nes-cre)1Kln/J (The Jackson Laboratory, #003771); B6.Cg-Tg(Vav1-icre)iKio (The Jackson Laboratory, #008610); B6.Cg-Tg(Cd4-cre)1Cwi/BfluJ (The Jackson Laboratory, #017336); Ncr1Cre mice were kindly provided by E. Vivier. Tg(Plp1-cre/ERT)Pop107 (The Jackson Laboratory, #005975).<br>All transgenic mouse strains were bred in-house and C57BL/6 wild type mice were purchased from Janvier. Age- and sex-matched (male and female) 8 to 12 week-old mice were used for all experiments. All animals were housed in conditions of 22°C and 45-65% humidity with 12 hours light cycle.
Wild animals	not used
Reporting on sex	Both male and female mice were used for experiments.
Field-collected samples	not used
Ethics oversight	All procedures were reviewed and approved by the Swiss Veterinary Office and performed according to institutional and federal guidelines.

Note that full information on the approval of the study protocol must also be provided in the manuscript.

Flow Cytometry

Plots

Confirm that:

☐ The axis labels state the marker and fluorochrome used (e.g. CD4-FITC).

☒ The axis scales are clearly visible. Include numbers along axes only for bottom left plot of group (a 'group' is an analysis of identical markers).

☐ All plots are contour plots with outliers or pseudocolor plots.

☒ A numerical value for number of cells or percentage (with statistics) is provided.

Methodology

Sample preparation	Isolation of immune cells and neural cells from the adult mouse CNS : Mice were sacrificed through CO_2 asphyxiation and transcardially perfused with ice-cold PBS. Whole brain and spinal cord were isolated, cut into small pieces and digested with 0.4 mg/ml Collagenase IV (#9001-12-1, Sigma-Aldrich) and 0.2 mg/ml deoxyribonuclease I (DNase I) (#E1010, Luzerna) in HBSS (with Ca^{2+} and Mg^{2+}) (#14025-050, Gibco) for 40 min at 37°C. The digested tissue was then mechanically dissociated using a 19-gauge needle and filtered through a 100 µm cell strainer (800100, Bioswisstec). CNS single-cell suspensions were further enriched by 30 % Percoll® (P4937, GE Healthcare) gradient centrifugation (1590 g, 30 min, at 4°C, with no brakes).<br>To enrich for and improve oligodendrocyte viability for downstream FACS-sorting and RNA isolation CNS tissue was subjected to enzymatic digestion using papain (#LS003126, Worthington) for 30 min at 37°C and the enzymatic reaction stopped with ovomucoid trypsin inhibitor (#LS003086, Worthington). Myelin debris was removed by 30% Percoll® (P4937, GE Healthcare) gradient centrifugation.<br><br>Single nuclei suspensions: Mouse cerebellum and brainstem (of one brain hemisphere) and cervical spinal cord (C1-C2) were harvested from adult male mice (8-12 weeks old) and immediately snap-frozen in liquid nitrogen and stored at -80oC until further processing. Nuclei were isolated with the EZ PREP lysis buffer (#NUC-101, Sigma). Tissue samples were homogenized using a glass dounce tissue grinder (#D8938, Sigma) (25 strokes with pastel A, 25 strokes with pastel B) in 2 ml of ice-cold EZ prep lysis buffer and incubated on ice with an additional 2 ml of ice-cold EZ PREP lysis buffer. During incubation, 1 µM of Hoechst (#H3570, Thermo Fisher Scientific) dye and 40 U/µl of RiboLock inhibitors (#EO0382, Thermo Fisher Scientific) were added to the homogenate. Following incubation, the homogenate was filtered through a 30 µM FACS tube filter. Nuclei were sorted based on the fluorescent Hoechst signal using a BD FACS Aria sorter III with an 85 µm nozzle configuration at 4oC, directly into PBS + 4% BSA + RiboLock inhibitors (40 U/µl) (Cat # EO0382).<br><br>Isolation of adult murine splenocytes: Spleens from steady-state mice were directly mashed through a 70 µm filter, without any prior digestion. Cells were washed once with 1x PBS (1500 rpm, 10 min, at 4°C) and the cell pellet was resuspended in RBC lysis buffer (NH_4Cl 8.3 mg/mL, $KHCO_3$ 1.1 mg/mL, EDTA 0.37 mg/mL) to lyse erythrocytes. After lysis, cells were washed once and re-filtered.
Instrument	Spectral flow cytometry: 5L Cytek® Aurora; SpectroFlo® v2 Software;<br>Fluorescence Activated Cell Sorting: BD FACSAria™ III

Software	Spectral flow cytometry: SpectroFlo® v2 Software;<br>Fluorescence Activated Cell Sorting: BD FACSDIVA Software
Cell population abundance	Abundance is reported in Figures 1, 3 and 4 and Extended Data Figure 3. Sorting was performed with the "Purity" mask, thus purity of all sorted populations is >95%.
Gating strategy	Spectral flow cytometry: cells were gated on FSC-A and SSC-A to remove debris; doublets were excluded by FSC- Area vs. FSC-Height gating. Dead cells were excluded using a Fixable Viability Kit (Near-IR staining, Biolegend). We manually pregated on single, live CD45+ cells and proceeded with high dimensional data analysis in R studio, as described above.<br><br>For manual gating of bone marrow chimeras data we applied the following gating strategy for the depicted populations:<br>CD4+ T cells: CD45+ CD44+ CX3CR1- LY6G- LY6C- CD11b- TCRbeta+ CD8- CD4+<br>Monocyte derived cells (MdCs): CD45+ CD44+ CX3CR1- LY6G- TCRbeta- NK1.1- SiglecF- CD11b+ LY6C+MHCII+<br><br>Single nuclei suspensions: As CNS nuclei vary strongly in size, no doublet exclusion was performed based on FSC or SSC to avoid bias against nucleus size. Nuclei were sorted based on the fluorescent Hoechst+ signal.<br>- neuronal nuclei: Hoechst+NeuN+ nuclei were sorted without doublet exclusion<br>- oligodendrocyte nuclei: Hoechst+Olig2+ were sorted without doublet exclusion<br><br>RT-qPCR data: Gating strategies for sorting of CNS resident cell populations (assessing targeting efficiency and specificity or Il12rb1 and Il12rb2 expression) are not shown, but described in the respective figure legends.<br>- immune cell populations: we pregated on single, live CD45+ cells<br>- neuroectodermal CNS populations: we pregated on single, live, CD45- cells<br><br>We applied the following gating strategy to determine the NK cell depletion efficiency (we pregated on single, live cells)<br>- CNS: NK cells (CD45+CD4-CD8-CD3-B220-Ly6G-CD44+CXCR1-CD49b+)<br>- Spleen: NK cells (CD45+CD4-CD8-CD3-B220-Ly6G- CD49b+)<br>Gating strategies are not shown, but provided in the respective figure legends

☐ Tick this box to confirm that a figure exemplifying the gating strategy is provided in the Supplementary Information.
