# Supplementary figures and images for: IL-12 sensing in neurons induces neuroprotective CNS tissue adaptation and attenuates neuroinflammation in mice
Source: Nat Neurosci. 2023 Sep 25;26(10):1701–12. doi: 10.1038/s41593-023-01435-z (PMC10545539; doi:10.1038/s41593-023-01435-z)

Neuronal culture

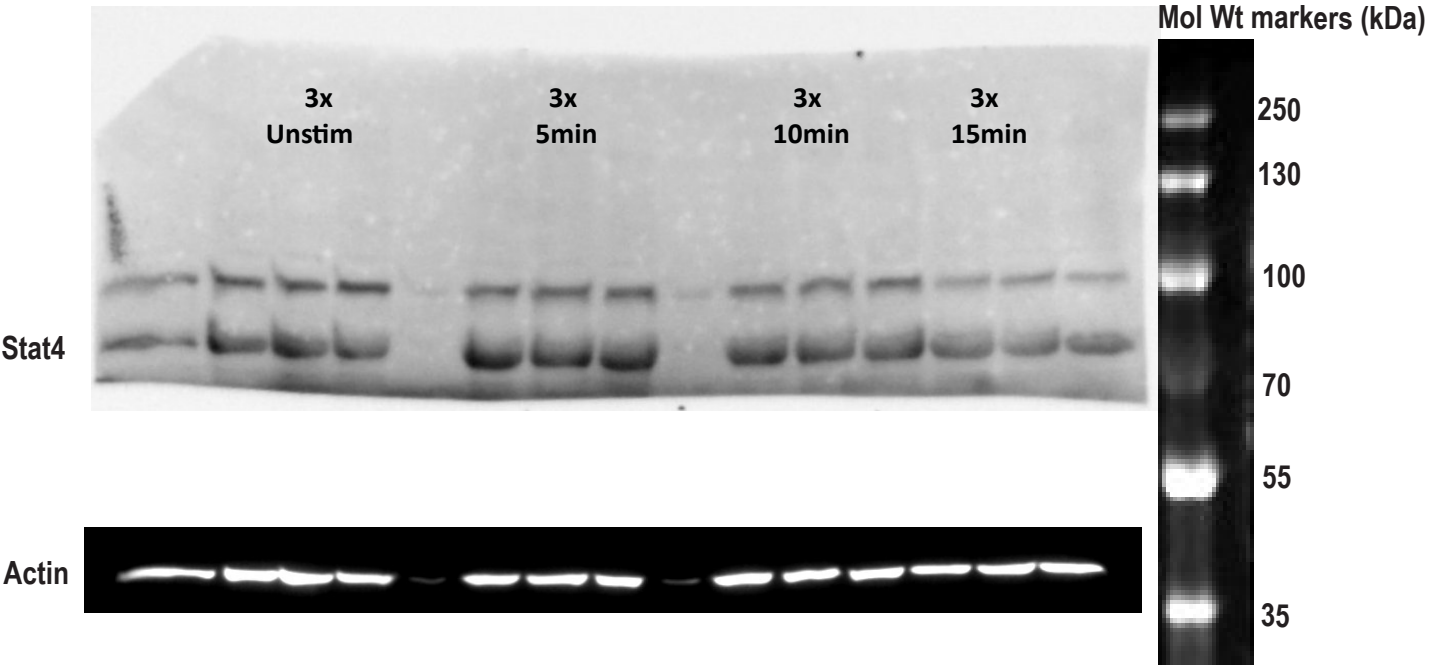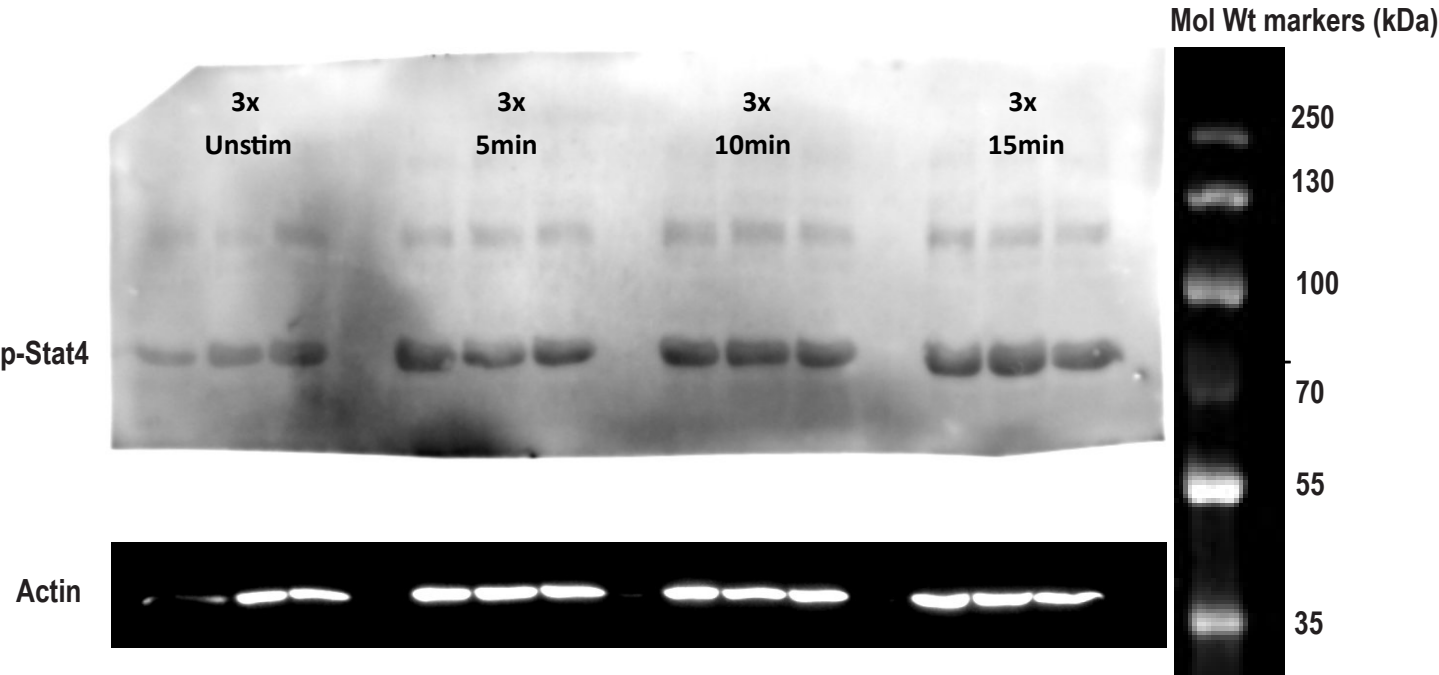

Oligodendrocyte culture

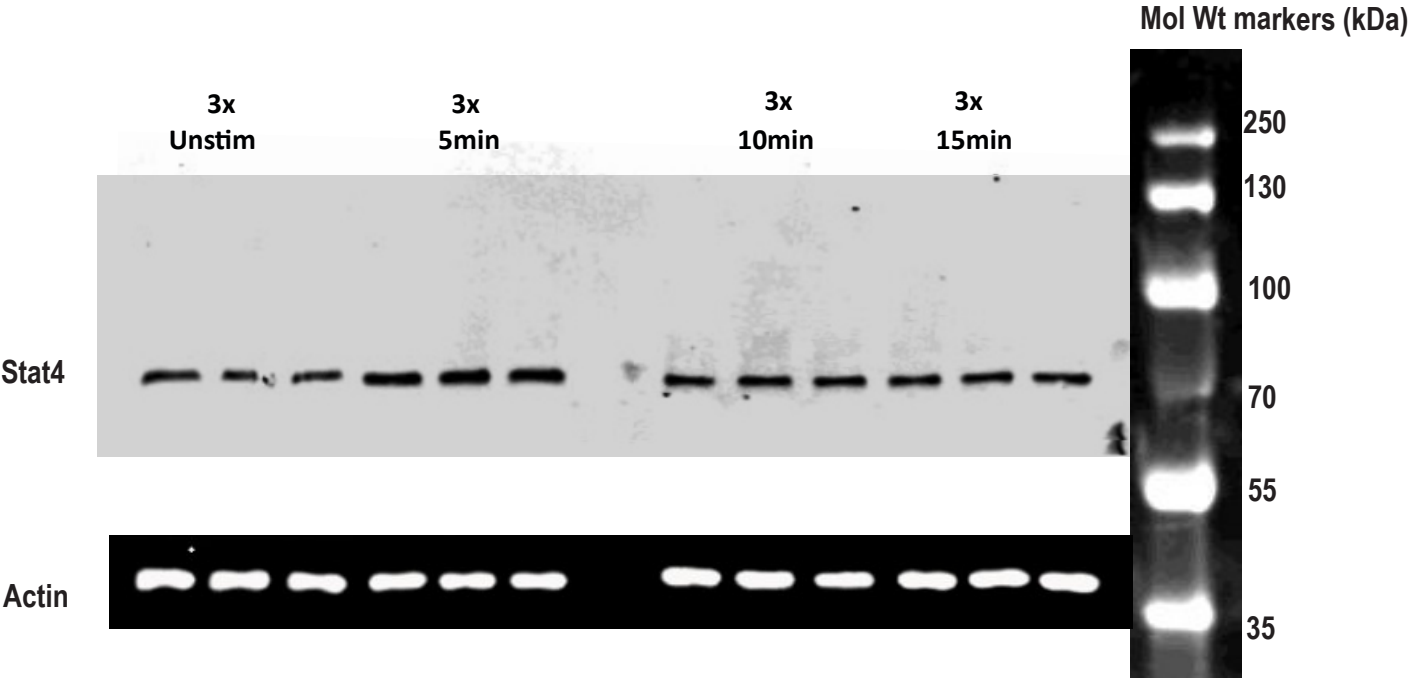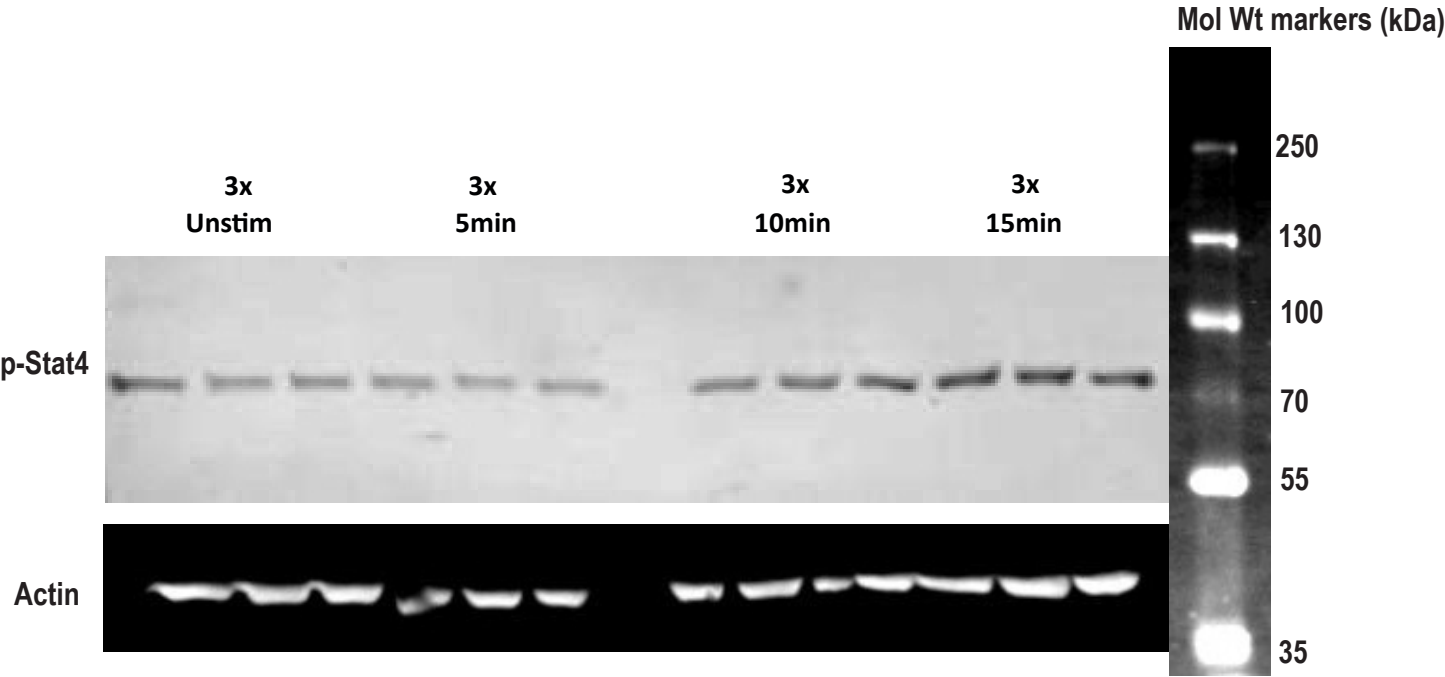

Supplement: Supplementary file 11 — Full-length, unprocessed gels or blots. [file 41593_2023_1435_MOESM11_ESM.pdf]
